# Metabolic Task Analysis Reveals Distinct Metabotypes in End-Stage Dilated Cardiomyopathy

**DOI:** 10.1161/CIRCGEN.125.005366

**Published:** 2026-07-03

**Authors:** Bastien S.C. Nihant, Job A.J. Verdonschot, Sonia Bălan, Eva Thielecke, Joost J.F.P Luiken, Miranda Nabben, Stephane Heymans, Marian Breuer, Michiel E. Adriaens

**Affiliations:** 1 Cardiovascular Research Institute Maastricht (CARIM), The Netherlands (B.S.C.N., J.A.J.V., J.J.F.P.L., M.N., S.H.).; 2 Maastricht Center for Systems Biology and Bioinformatics (MaCSBio), Maastricht University, The Netherlands (S.B., E.T., M.B., M.E.A.).

**Keywords:** amino acids, calcium, cardiomyopathy, dilated, gene expression profiling, heart failure, humans, inflammation

## Abstract

**BACKGROUND:**

Dilated cardiomyopathy (DCM) is associated with shifts in cardiac metabolism. However, those shifts vary widely across patients, likely reflecting the diverse underlying causes of the disease. Identifying metabolic subtypes, or metabotypes, in DCM patients could help tailor treatments to patient needs. Hence, having a practical approach to identify these metabotypes would be a significant advance toward precision medicine in DCM.

**METHODS:**

We present a systems biology approach to uncover metabotypes directly from widely available transcriptomic data. We use in silico metabolic modeling methods that we have optimized for cardiac research to predict metabolic function activities from enzyme expression, followed by a hierarchical clustering approach. To demonstrate its power, we applied our method to publicly available cardiac data from end-stage DCM patients (N=164) and nonfailing controls (N=160).

**RESULTS:**

We identified 2 distinct metabotypes in end-stage DCM patients. These metabotypes are characterized by unique metabolic changes, notably in calcium handling, amino acid oxidation, and the pentose phosphate pathway. Strikingly, 1 DCM metabotype showed greater metabolic divergence from healthy controls, suggesting a greater metabolic contribution to its underlying etiology—even though disease severity was similar between the 2 identified DCM metabotypes. Further transcriptome-wide analysis revealed immune-related differences between metabotypes, suggesting an underlying interplay between inflammation, the immune response, and metabolism.

**CONCLUSIONS:**

Our results imply the presence of distinct metabotypes in end-stage DCM. Our systems biology approach offers an exciting opportunity to uncover novel insights into DCM, paving the way for a deeper understanding of its progression and heterogeneity.

The heart is one of the most energy-demanding organs in the body, estimated to consume 400 kcal/d, or 15% to 20% of total caloric usage.^1^ To sustain its constant activity, it has evolved to use various nutrients, a concept known as metabolic flexibility. Fatty acids in particular represent about 60% of substrate usage in the healthy heart.^2^ Dysregulation of cardiac substrate usage has been observed in heart failure (HF). More specifically, there is a shift away from fatty acid oxidation and toward glycolysis.^3^ This has also been observed in dilated cardiomyopathy (DCM), a nonischemic form of HF.^4^ These metabolic changes, however, have recently been under some scrutiny. Targeting metabolism as a treatment strategy has proven to be difficult.^5^ While, for example, sodium-glucose cotransporter-2 inhibitors have shown benefits in DCM treatment, their effect is much broader than glucose metabolism, and it is still unclear through which mechanisms they benefit DCM patients.^6^ Furthermore, recent studies have challenged the extent of loss of metabolic flexibility in nonischemic HF.^7^ Further research in this field is therefore needed to provide more definitive answers.

An important consideration is that DCM should be considered a phenotype arising from various genetic and nongenetic disease triggers. Even prevalent DCM-related variants, such as truncating titin variants, require environmental factors to trigger the phenotype, and many cases are still idiopathic.^8^ For this reason, it is accepted that the molecular profile of the DCM heart varies between patients. Previous studies showed distinct transcriptomic and metabolic profiles correlating with truncating titin variants, and with disease severity in nongenetic DCM patients.^9,10^ We therefore postulate that cardiac metabolism may be impacted differently between patients irrespective of severity, possibly explaining the lack of success of broadly metabolism-oriented treatments.^5^ Such differences between metabolic profiles are often referred to as metabotypes. This concept is particularly useful in personalized nutrition and medicine when tailoring treatment and recommendations to individuals.^11^ Different underlying metabotypes could in part explain the heterogeneity in results from studies of DCM cardiac metabolism.

Studying metabolic changes in human subjects is, however, complicated and costly.^12^ The gold standard for quantifying metabolic changes is metabolic flux measurement, using isotopic tracers such as ^2^H and ^13^C followed by mass spectrometry. Such flux analysis reliably measures metabolic conversions by following the fate of ingested or injected isotopes.^13^ Recent advances have enabled the use of magnetic resonance spectroscopy to infer metabolic rates, enabling noninvasive studies of specific metabolites in living patients.^14^

These methods are invaluable for investigating existing hypotheses. However, in some cases, being able to rapidly and cheaply generate insights from widely available data would be of significant value. Systems biology approaches are invaluable for such purposes. Genome-scale metabolic models (GEMs) are one such approach.^15^ GEMs consist of a network of reactions and metabolites, aiming at completely covering an organism’s metabolism with accurate stoichiometry. Reactions are modeled using steady-state fluxes representing the homeostatic reaction activity over time. Such steady-state flux-based modeling therefore simplifies the dynamics of metabolism in exchange for allowing modeling of the complete metabolism. Multiple types of methods exist to derive subject-specific insights from these generalized models.^16^

In this article, we refined an approach to directly infer metabolic functionality by combining a comprehensive human GEM and subject-level omics data.^17^ This approach is referred to as metabolic task analysis. Metabolic tasks describe net conversions of biological interest, such as the oxidation of glucose with oxygen to CO_2_ and water with production of ATP, defined in terms of their substrates’ and products’ stoichiometries. The metabolic task activity is then evaluated from the expression of the enzymes catalyzing the reaction pathways from substrates to products. In this way, metabolic task analysis queries the transcriptomic data for specific metabolic functions.^18^ This is particularly valuable, as it unlocks new metabolic insights specifically from existing transcriptomic data, one of the most readily available and widely utilized data types in molecular cardiology.

The generated task activity scores represent a detailed metabolic fingerprint and can be used for group comparisons and, importantly, metabotyping. It offers important advantages over traditional methods such as pathway enrichment analysis, which only indicates whether a pathway contains more differentially expressed genes than expected by chance. How to subsequently interpret enrichment results is not straightforward. In contrast, metabolic task analysis identifies bottlenecks in pathways and provides direct activity scores with a clear biological implication. Current implementations of metabolic task analysis, however, have limitations in their applicability to cardiac transcriptomic data. Namely, these tools are based on older GEMs and do not offer tasks related to myosin contraction or calcium handling in the sarcoplasmic reticulum (SR), hampering their application to the study of cardiac metabolism.^17,19,20^

Here we present a cardiac-specific metabotyping pipeline. We use the most recent human GEM and a list of metabolic tasks, both extended to cover cardiac-specific functionalities.^17,19,21^ We then use our pipeline on publicly available cardiac transcriptomic data from DCM subjects and healthy controls, with the hypothesis that distinct metabotypes would be apparent in DCM subjects. We not only uncover distinct metabotypes but also identify specific metabolic processes pointing to underlying mechanisms distinguishing them and provide targets for more tailored intervention strategies.

## Methods

This article utilized publicly available data from the Genotype-Tissue Expression (GTEx) project and Myocardial Applied Genomics Network (MAGNet) project. The data sets and related metadata from GTEx and MAGNet can be obtained from the portal (https://gtexportal.org/home/downloads/adult-gtex/bulk_tissue_expression) and GitHub repository (https://github.com/mpmorley/MAGNet), respectively. As only publicly available data were utilized in this project, institutional review board approval was not required. The scripts and other resources used during the current study can be found on the dedicated GitHub repository (https://github.com/Bastien-N/MetabolicTasksDCM).

The described analysis was performed using R, version 4.2.1, unless stated otherwise.^22^ A schematic representation of the metabotyping pipeline is provided in Figure S1.

### Validation RNA-Sequencing Data Processing

RNA-sequencing raw-count data from the GTEx project (version 8) were downloaded from the GTEx Portal on November 25, 2022. Samples from subcutaneous adipose tissue, transverse colon, cardiac left ventricular, renal cortex, liver, and skeletal muscle (gastrocnemius) were kept for further analysis (Supplemental Methods). The raw count data were normalized using geTMM (Gene length corrected trimmed mean of M-values) normalization,^23^ followed by batch effect correction using the Limma R package.^24^ As the metabolic task analysis method used, CellFie,^17^ includes logarithmic transformations internally, we shifted every value by 1 to ensure a minimum log-transformed value of 0.

### DCM RNA-Sequencing Data Processing

The MAGNet RNA-sequencing raw-count data (Gene Expression Omnibus accession number GSE141910) were downloaded from the consortium’s GitHub repository (https://github.com/mpmorley/MAGNet). We only included subjects with a DCM diagnosis or a nonfailing heart, as determined by the original authors.^4^ In addition, patients with a reported height under the usual definition of dwarfism (142 cm), although likely due to a reporting error, were excluded. The raw count data for the remaining 324 subjects (Table S1) were then processed as for the GTEx data.

### Metabolic Model and Task Analysis

All metabolic task analyses were performed using MATLAB, version 2021b and the Cobra Toolbox, version 3.1.^25,26^ We used the Human-GEM genome-scale metabolic model version 1.13.0, modified to fit the Cobra Toolbox conventions.^19,21^ We further modified the model by adding reactions representing the activities of the RyR sarcoplasmic calcium channels and the sarcoplasmic Ca^2+^ ATPase (Table S2). We curated a list of metabolic tasks by combining previously published lists,^17,19,20^ modified for use with our model, as well as tasks related to cardiac-specific function. Tasks expected not to occur in heart tissue were used as negative controls (Supplemental Methods). From this task list, essential reactions (ERs) were determined using the algorithm from the checkMetabolicTasks function of the Cobra Toolbox, with minor modifications (Table S3). This function checks whether the model contains the minimal set of reactions to complete each metabolic task, and ERs are metabolic chokepoints that must absolutely be active for the task to be completed. These ERs and the gene expression data were used in the CellFie function to produce task activity scores and binary task activity scores.^17^ In short, these scores are calculated from the average expression of enzymes contributing to ERs, weighted based on enzyme promiscuity. Although derived from gene expression data, task activity scores are best viewed as having arbitrary units and should not be directly compared across tasks. Binary scores define tasks as active if the activity score is higher than a set threshold. We then conducted 3 separate CellFie analyses. First with the GTEx data, second with all control and DCM samples of the MAGNet data, and finally with only the DCM samples of the MAGNet data (see next section). Tasks that failed the checkMetabolicTasks run (no feasible set of reactions to fulfill the task to begin with), or whose ERs were not related to any gene, such as those involved in passive transport reactions, were removed before further analysis. As inspired by the original CellFie paper,^17^ mixed task scores were created for each of these runs by multiplying the task scores by the binary task scores.

### Clustering Based on Task Scores

The described analysis was performed using R, version 4.2.1.^22^ Tasks considered active or inactive in all samples were excluded from clustering to help contrast samples. Principal component analysis was used to recover all principal components for the mixed task activity scores of the GTEx data explaining over 99.9% of the variance. We then clustered the samples with hierarchical clustering using Ward.D2 linkage based on these principal components. The appropriate number of clusters was determined via bootstrapping using the algorithm implemented in the fpc package version 2.2-11.^27^ In short, the algorithm performs an initial hierarchical clustering with all the samples for different numbers of clusters (k) from 2 to 15. Then, 10 000 bootstraps are performed in which subjects are randomly sampled and re-clustered. For each value of k, the Jaccard similarity index between clusters in the original data and the closest clusters in the resampled data is calculated (see the fpc package documentation for further details). We considered clusters to be stable if they obtained a mean Jaccard similarity above 0.6 across bootstraps. Clusters obtained were assessed visually using a heatmap and numerically by calculating their purity, that is the percentage of samples belonging to the most common tissue type in the cluster.

Based on the glycerol-3-phosphate synthesis, GTEx results, we applied the same clustering pipeline to the metabolic task activity of DCM samples in the MAGNet data set. For this analysis, we only included DCM samples when calculating activity scores. This was done to avoid tasks being considered inactive in all DCM samples due to lower activity scores in all DCM subjects relative to controls, which would affect gene activity thresholds calculated internally. Such tasks may be meaningfully different between DCM subjects and therefore be relevant for identifying subtypes.

### Hypothesis Testing

We performed Mann-Whitney *U* tests between tissue types for the GTEx-derived task activity scores of a selected set of metabolic tasks. We selected these tasks (*aerobic rephosphorylation of ATP from glucose*, *glycerol-3-phosphate synthesis*, and *fructose-to-glucose conversion*) due to their having predictable differences between tissues based on previous literature and databases.^1,28–30^

We used the Mann-Whitney *U* test for every task to determine whether the task activity scores were significantly different between (1) controls and DCM subjects, (2) controls and DCM subjects from each metabotype, and finally (3) the 2 identified metabotypes. The first 2 analyses were performed using scores calculated using controls and DCM samples, and the third analysis was performed using scores calculated only using DCM samples. Results are provided for every task, although we focused our interpretation on selected tasks of interest. These tasks were selected as being readily interpretable from the task’s ERs and as being relevant to cardiac energy metabolism. The chosen tasks cover pathways related to energy generation, amino acid (AA) metabolism, and cardiac-specific functions (Table S4). In addition, tasks that were considered inactive were not included, as their score would have derived from genes expressed at a level we would mostly consider to be noise. To assess the possible confounding influence of age, sex, and ethnicity in the comparisons between controls and metabotypes, we used ordinal logistic regression models. In these models, the activity score rank was used as the outcome, with group, age, sex, and ethnicity as predictors. We used nonparametric statistical tests as we found task activity to typically violate the normality assumption of *t* tests (Figure S2). In addition, we compared the metabotypes in terms of the limited personal and clinical information available to us (age, sex, body mass index, ethnicity, diabetes, hypertension, and left ventricular ejection fraction) using Pearson χ^2^ tests and the Mann-Whitney *U* test, as appropriate.

### Immune Cell-Type Deconvolution

We applied the CIBERSORTx pipeline (run from https://cibersortx.stanford.edu),^31^ combined with its publicly available immune cell signature matrix, to the MAGNet data set. For this purpose, the data were normalized into fragments per kilobase of transcript per million. We compared abundance percentages between controls and metabotypes, as well as between metabotypes, using the Mann-Whitney *U* test, corrected for multiple testing with the Benjamini-Hochberg procedure.

### Differential Gene Expression Analysis

Differential gene expression analysis was performed using the edgeR R package, version 4.0.3.^32^ The gene expression data were filtered to remove lowly expressed genes using the default parameters of the filterByExpr function. After calculating trimmed mean of M values normalization factors, a quasi-likelihood negative binomial generalized log-linear model was fitted to the data. The model included the experimental batches and the group variable describing subjects as controls or members of a DCM cluster. We then performed differential gene expression analysis using the glmTreat function, contrasting control subjects with subjects from each cluster, as well as contrasting cluster subjects. For more stringent results, a minimal log_2_-fold change of log_2_(1.5) was required. We used the clusterProfiler package, version 4.10.0, to perform gene set enrichment analysis using gene ontologies for the tested contrasts.^33–35^ The analysis comparing controls with each metabotype was then repeated while including age, sex, and ethnicity as covariates.

## Results

### Metabolic Task Analysis Captures Metabolic Differences Between Tissues

**Figure 1.**
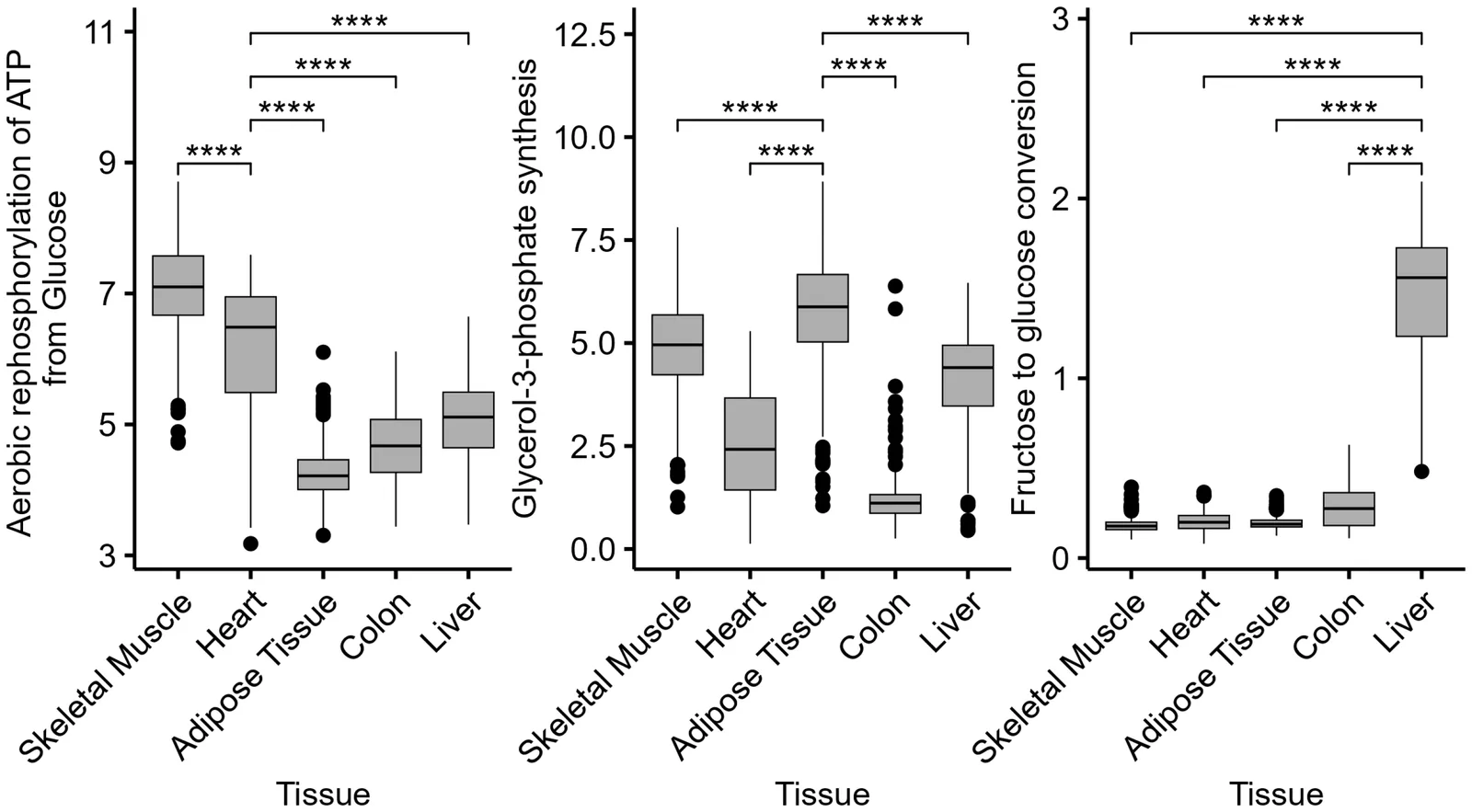
**Metabolic task activity captures differences between human tissues metabolic functions.** Boxplots of task activity scores (arbitrary units) for 3 representative tasks, grouped by tissue in the Genotype-Tissue Expression data. ****P<0.0001.

We first tested the pipeline’s ability to pick up differences between metabolically distinct tissues (subcutaneous adipose, transverse colon, cardiac left ventricular, skeletal muscle, and liver) by clustering the GTEx samples based on task activity. In doing so, we produced 7 tissue specific clusters, although some tissues were split into multiple clusters (average purity of 96%; Figure S3). As expected, we observed that the energy-demanding cardiac and skeletal muscle tissues presented with significantly higher activity for the *aerobic rephosphorylation of ATP from glucose* task (Figure 1, left).^1,28,29^ Adipose tissue samples had the highest task activity for the *glycerol-3-phosphate synthesis* task (Figure 1, middle).^28^ Finally, liver tissue samples were the only ones in which *fructose-to-glucose conversion* appeared active (Figure 1, right), as expected from mRNA-derived values.^30^ Colon tissue showed consistently low activity scores for these tasks relative to other tissues.

### End-Stage DCM Patients Separate into Two Distinct Metabotypes

**Figure 2.**
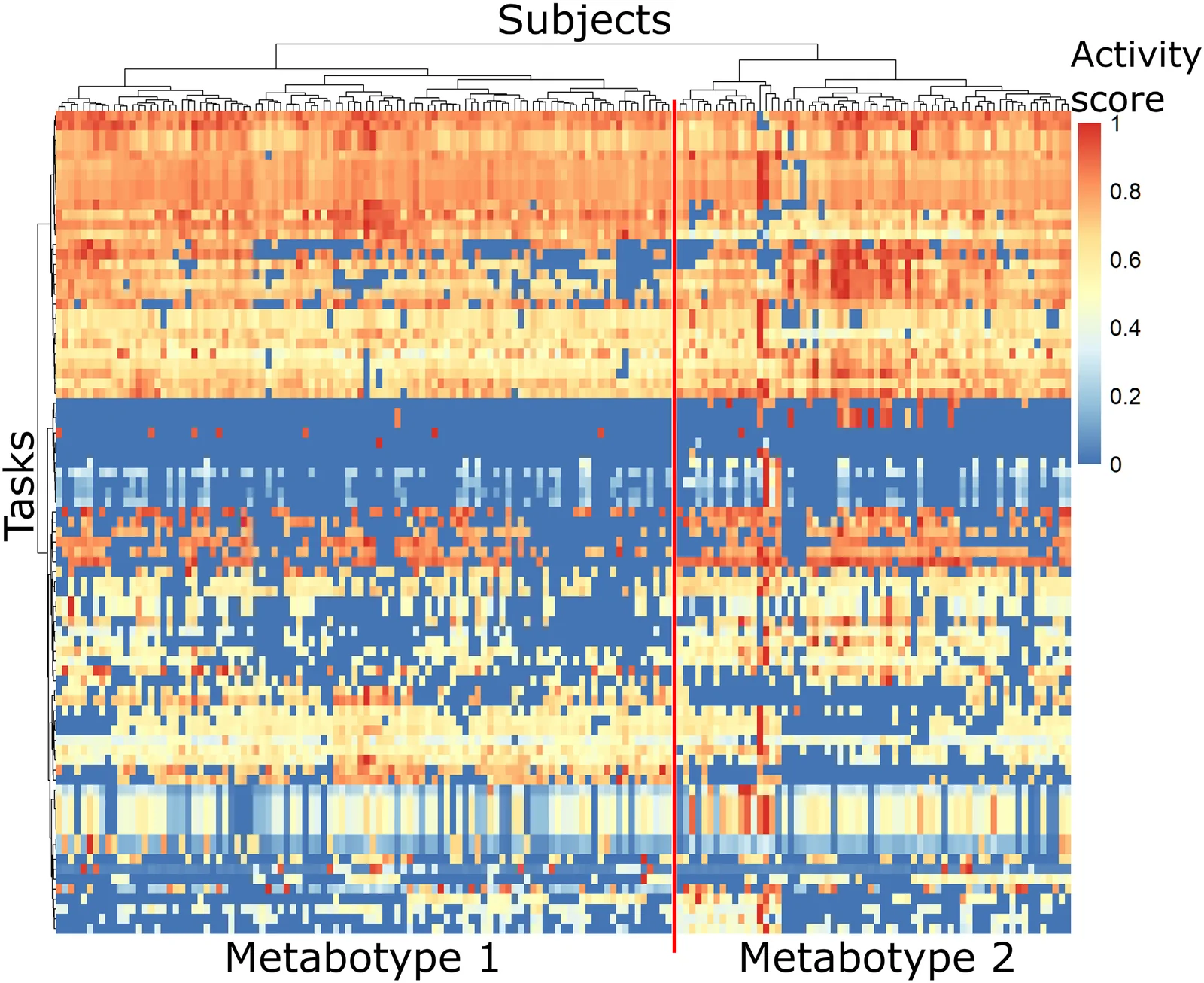
**Clustering outlines 2 metabotypes based on metabolic task activity.** Heatmap of scaled (min-max scaling) mixed task scores of dilated cardiomyopathy subjects for the tasks included in clustering with cluster separation indicated by a red line.

Next, we applied our metabolic task analysis pipeline to a publicly available RNA-sequencing data set of left ventricular biopsies from 164 DCM patients and 160 nonfailing controls (Table S1). To assess whether subgroups exist within the DCM subjects, we applied our pipeline to the DCM samples separately and identified 2 stable clusters, from here on referred to as metabotype 1 and metabotype 2 (Figure 2; Jaccard coefficients of 0.67 and 0.64, respectively, relative to their closest cluster in each bootstrapped clustering run).

The generated metabotypes presented with distinct metabolic phenotypes based on metabolic task analysis. After accounting for multiple testing, 48 tasks had significantly different activity between metabotypes with a false discovery rate under 0.05 and an absolute fold change of 10% (Table S5). Comparisons were also made between control subjects and each metabotype, with 43 tasks being significantly altered in metabotype 1 and 54 tasks altered in metabotype 2 (Table S5). The top 10 significantly different tasks were largely similar for all comparisons involving control subjects, with the task of *carnosine synthesis from β-alanine and histidine* being the most upregulated in all DCM metabotypes (Figure S4). By focusing our analysis on predefined tasks of interest, we were able to uncover the distinguishing features that define the metabotypes (Table 1 and Figure S5).

**Table 1.**
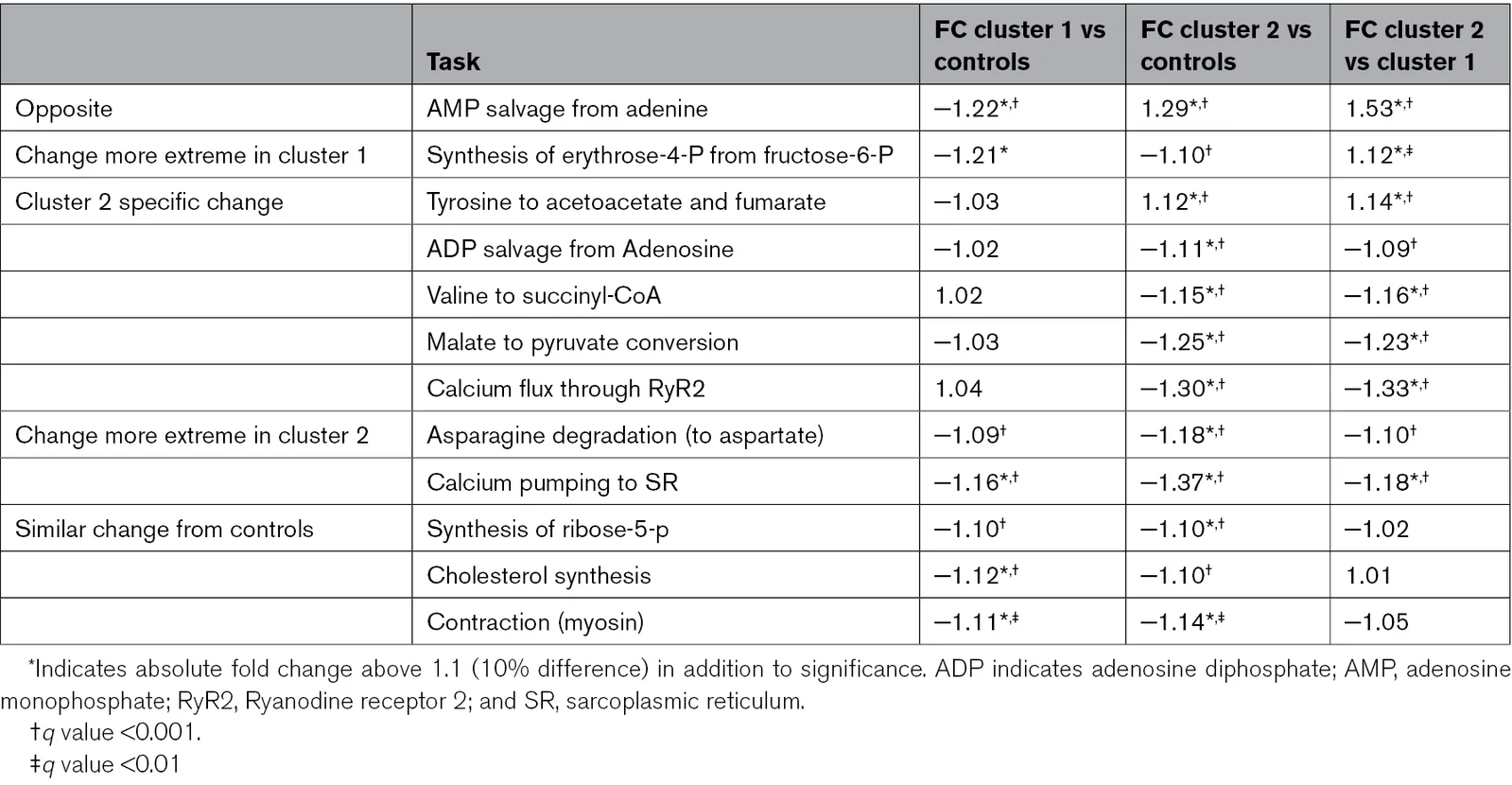
Fold Changes of Tasks of Interest Significant in at Least 1 Comparison

Cardiac-specific tasks were significantly affected in both metabotypes, with downregulation of myosin contraction and calcium pumping into the SR. Notably, the latter was further suppressed in metabotype 2, along with *calcium flux through RyR2*. Tasks related to the pentose phosphate pathway (PPP), ribose-5-phosphate and erythrose-4-phosphate synthesis, were downregulated in DCM, the latter especially in metabotype 1. AA metabolism was especially affected in metabotype 2, with higher activity of *tyrosine to acetoacetate and fumarate* and lower activity of *valine to succinyl-CoA*. Asparagine degradation was inhibited in both groups but especially in metabotype 2. AMP salvage was downregulated in metabotype 1 and upregulated in metabotype 2, where ADP salvage was lower than in controls. Finally, *malate-to-pyruvate conversion* was uniquely downregulated in metabotype 2 while cholesterol synthesis was inhibited in both metabotypes (Table 1). Adjusting for age, sex, and ethnicity did not lead to meaningful differences in the results (Figure S6).

We did not observe significant differences between metabotypes in terms of the limited available phenotypic and clinical information, including age, sex, ethnicity, body mass index, diabetes and hypertension diagnosis, or left ventricular ejection fraction. Median left ventricular ejection fraction was 15% in both metabotypes, consistent with the end-stage characterization of these subjects (Table 2).

**Table 2.**
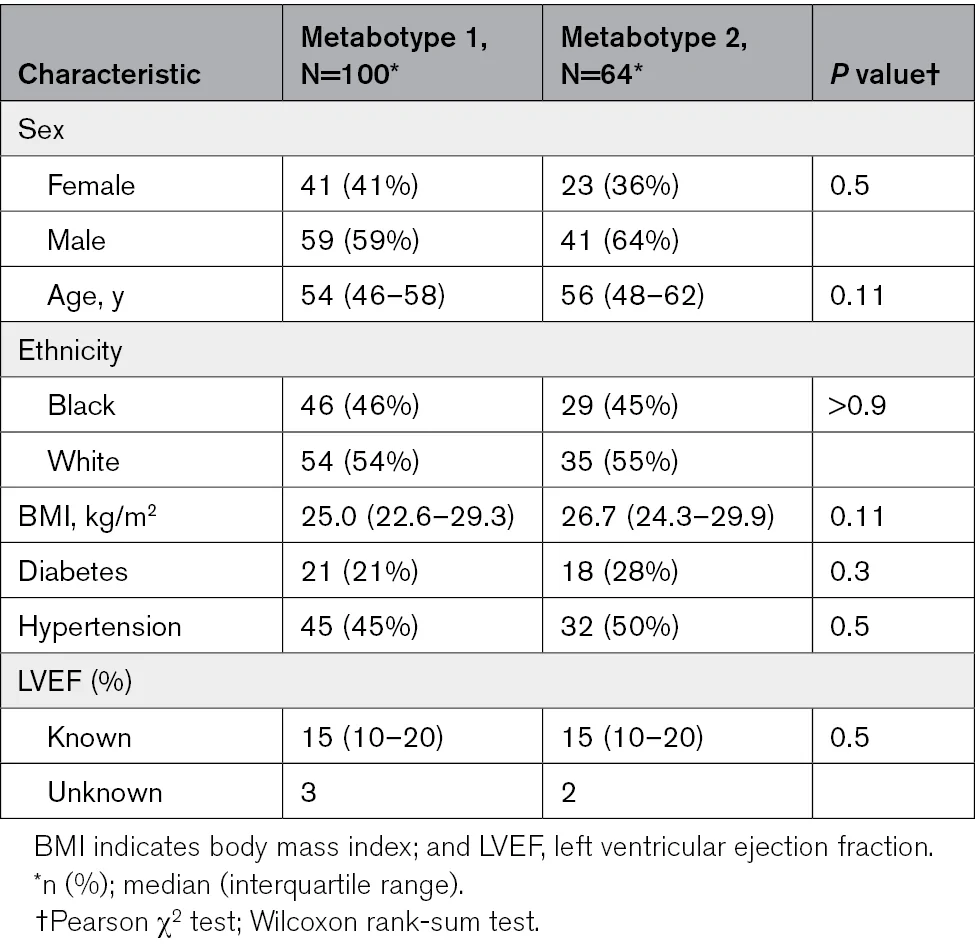
Summary Characteristics of Dilated Cardiomyopathy Subjects by Metabotype

### Differential Expression Analysis Suggests a Relationship Between Cardiac Metabotypes and the Immune System

**Figure 3.**
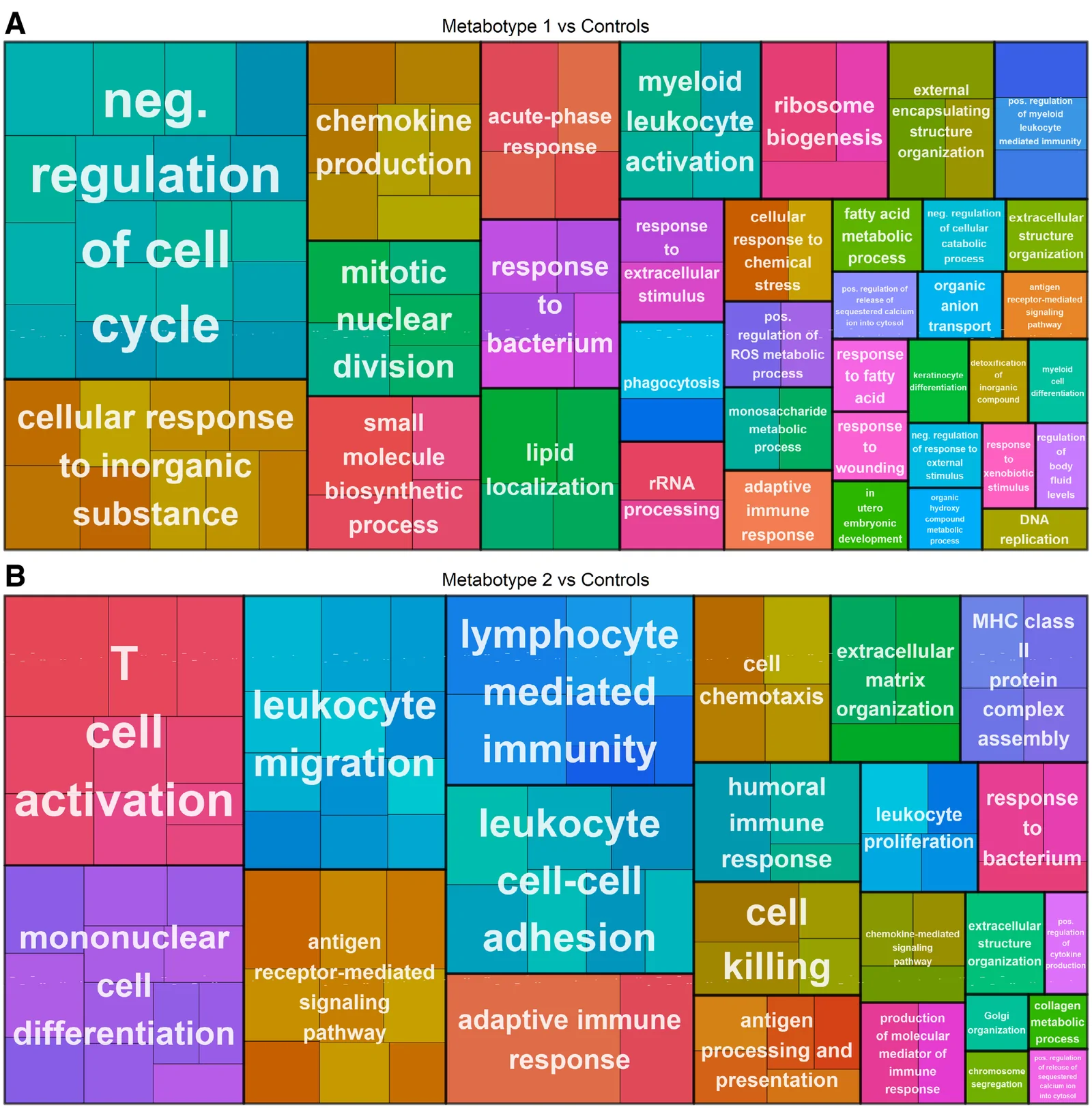
**Broad transcriptomic differences are present between control samples and each metabotype.** Treemap plot of top 100 significantly enriched gene ontologies between controls and metabotypes 1 (**A**) and 2 (**B**), grouped by similarity and represented by the most significant ontology in each group. Small rectangles represent individual ontologies with area relative to gene set enrichment analysis (GSEA) score. Colors assigned at random for visual contrast.

Next, we extended the analysis of the metabotypes to the whole-transcriptome level by including differential expression analysis to assess differences beyond metabolism. We found 1336 and 2968 differentially expressed genes when comparing controls with metabotype 1 and metabotype 2, respectively (adjusted *P*<0.05 and absolute FC >1.5; Figure S7). Comparing controls with metabotypes revealed many enriched lymphocyte-related gene ontologies in metabotype 2, while metabotype 1 presented more enriched ontologies related to cell-cycle regulation and chemokine production (Figure 3). A total of 1317 genes were differentially expressed between metabotypes, with mostly immune-related ontologies being enriched (Figure S8). Adjusting for age, sex, and ethnicity did not change the outcome of the analysis (Figures S9 and S10).

### Cell-Type Deconvolution Suggests Differences in Immune Cell Infiltration Between Metabotypes

To follow-up on these results, we imputed immune cell-type abundance in DCM samples using the CIBERSORT algorithm. Estimated abundance was significantly different between groups for multiple cell types (Figure S11). DCM subjects, regardless of metabotype, showed an increased proportion of γδ and CD8 T-cells, activated CD4 memory T-cells, activated dendritic cells, and B-cells. Furthermore, there were decreases in resting CD4 memory T-cells and memory B-cells. M2 macrophages were lower in DCM subjects as well, and even further reduced in metabotype 1. This metabotype was also characterized by a higher proportion of resting dendritic cells as well as lower monocytes. In metabotype 2, we found a higher abundance of activated natural killer cells and lower neutrophils. These results imply immune infiltration differences in DCM patients, most of them consistent between metabotypes.

## Discussion

### Summary of Main Findings

We constructed a metabotyping pipeline combining established methods and models^17,19,21^ which we refined to better suit cardiometabolic research by covering functionalities such as sarcoplasmic calcium handling. We demonstrated the effectiveness of our pipeline using GTEx reference RNA-sequencing data from different tissues. Accordingly, we showed that our updated pipeline is able to differentiate between tissues both through clustering and through hypothesis testing on tasks related to tissue-specific metabolic requirements. After having validated our pipeline, we applied it to publicly available data from end-stage DCM subjects and control nonfailing donors. By doing so, we uncovered 2 metabotypes in patients with end-stage DCM.

Notably, multiple tasks showed similar directional differences from controls for both metabotypes, even when the activity score was significantly different between metabotypes. Metabotype 1 is characterized by a downregulation of AMP salvage, coupled with a stronger downregulation of the PPP than metabotype 2. Metabotype 2, on the other hand, is characterized by changes in a wider range of tasks covering AA metabolism, portions of the Krebs cycle, and calcium handling-related tasks. Metabotype 2 may therefore represent patients in whom cardiac metabolism is more severely affected than in metabotype 1.

In addition to the observed effects on metabolic functionalities, differential gene expression analysis revealed differences in immune-related pathways: metabotype 1 is associated with cell-cycle inhibition and innate immune responses, while metabotype 2 shows more significant T-cell related pathways. These findings suggest that metabotype 2 may be more affected by immune-related dysregulation, potentially contributing to a more severe disease phenotype.

### Interpretation of Main Findings

Widespread differential expression in the cardiac transcriptomic profile was observed between controls and end-stage DCM subjects. Dividing the subjects into metabotypes helps define the heterogeneity in the metabolic phenotype displayed in the complete group of DCM patients. Both metabotypes exhibited reduced task activity for the *contraction* and *cholesterol synthesis* tasks. Previous research has shown that myosin light-chain kinase may be reduced in end-stage HF patients and in left ventricular assist device nonresponders,^36,37^ while reduced plasma cholesterol is associated with end-stage HF, although it is unclear how that would reflect in cardiac cholesterol synthesis.^38^ Similarly, *ribose-5-phosphate synthesis*, a step of the PPP, is downregulated in both metabotypes. The *synthesis of erythrose-4-P from fructose-6-P* task, an entry point into the PPP, is inhibited in both metabotypes, although to a greater extend in metabotype 1. PPP downregulation is in accordance with the observed reduction in PPP intermediate metabolites in the subset of these patients analyzed in Flam et al,^4^ possibly indicating that glucose is being redirected toward other pathways such as glycolysis.

Other tasks, such as *calcium pumping to the SR*, displayed a stronger reduction in metabotype 2 than in metabotype 1. In line with this observation, downregulation of sarcoplasmic/endoplasmic reticulum Ca2+ transporting 2, or SERCA2, the protein responsible for the active transport of Ca^2+^ into the cardiac SR, has been observed both at the mRNA and protein levels in ischemic HF and idiopathic DCM.^39,40^ Such a reduction in Ca^2+^ pumping may limit the SR Ca^2+^ concentration, thus inhibiting contractility through reduced Ca^2+^ release.^40^ This may be to a greater extent in metabotype 2 considering the task activity difference. In addition, the *calcium flux through ryanodine receptor 2 (RyR2*) task is significantly reduced in metabotype 2. RyR2 protein expression differences has been observed in HF.^41^ However, posttranscriptional modifications of RyR2, which we do not address in this study, have been more extensively related to HF by impairing calcium handling.^41^ We suspect that parallel dysregulation of calcium pumping into and release from the SR may contribute to calcium handling impairments, which may contribute to the HF pathology in metabotype 2.

Metabotype 2, compared with controls, shows changes in AA metabolism, including a decrease in asparagine and valine degradation and an increase in tyrosine degradation. Lower aspartate levels, a product of asparagine degradation, are linked to poorer HF prognosis.^42^ Branched-chain AAs like valine play a key role in heart function, with impaired valine oxidation possibly predicting major adverse cardiac events in nonischemic DCM.^43–45^ This study is the first, to our knowledge, to connect tyrosine metabolism to DCM, suggesting an increased demand for ketone bodies as a substrate, supported by previous mouse model research.^46^

Finally, AMP salvage from adenine was significantly different from controls in opposing directions in both clusters, being increased in metabotype 2 but reduced in metabotype 1. In addition, salvage of ADP from adenosine (with AMP as an intermediary product) was found downregulated in metabotype 2. The metabotype 1 results can be related to the 2022 paper by Flam et al,^4^ which used a subset of the MaGNet data set used here. They observed in DCM a downregulation of the adenine phosphoribosyltransferase (APRT) enzyme, responsible for the AMP salvage reaction, as well as a decrease of tissue adenine.

Our literature search uncovered no mention of cardiac APRT upregulation. Still, APRT upregulation does promote cutaneous wound recovery.^47^ A similar effect may occur in cardiac tissue repair, though more evidence is needed to support this hypothesis. This result may also reflect a compensatory effect in metabotype 2 instead.

Both AMP and ADP salvage relate to the purine salvage pathway, which is crucial to energy homeostasis in cells by helping maintain a proper ATP/AMP ratio. Dysregulation of this ratio can impair the balance of anabolic and catabolic metabolism through AMPK (AMP-activated protein kinase).^48^ AMPK-deficient mice models have been shown to develop DCM symptoms.^49^ Similarly, AMPK inhibition is postulated as a mechanism in doxorubicin-induced DCM.^50^ Conversely, AMPK activation is regarded as a beneficial therapeutic strategy in cardiac hypertrophy and ischemic heart disease.^51,52^ However, to our knowledge, the role of AMPK in idiopathic DCM is not quite established yet.

Together, results in tasks of interest imply that metabotype 2 differs more from controls relative to metabotype 1, possibly indicating a more severe HF. However, all DCM subjects were already classified as end-stage, requiring transplantation, and left ventricular ejection fraction was similar in both metabotypes. The hypothesized advanced metabolic phenotype of metabotype 2 is therefore more likely to reflect a difference in the combination of factors that lead to DCM, rather than disease severity.

Following the metabolic task analysis, we performed differential expression analysis and gene-set enrichment analysis to investigate whether metabotypes reflected differences in the whole transcriptome. Most of the metabolic particularities of metabotype 2 observed in the metabolic task analysis are not visible in this follow-up gene set enrichment analysis. We think that this highlights the usefulness of metabolic task analysis to detect effects that may go unnoticed in usual analysis.

The overwhelming majority of differential expression relates to immune and inflammatory genes. Metabotype 1 is characterized by ontologies related to cell-cycle inhibition and innate immune response. Cell-cycle regulation, specifically the Myc gene, has been implicated in various cardiomyopathy animal models and in hypertrophic cardiomyopathy, where it relates to a pathological fetal re-expression pattern.^53^ Metabotype 2, on the other hand, is heavily characterized by T-cell related ontologies.

As reported before, 9% to 50% of DCM cases involve cardiac inflammation, or myocarditis.^54^ The disease is suspected to progress from chronic inflammation to cardiac remodeling with the involvement of many immune cell types including T-cells.^55,56^ T-cell infiltration in the myocardium is a criterium for myocarditis diagnosis.^57–59^ Th17 CD4+ T-cells also promote myocarditis progression into DCM through IL-17A production.^60,61^ Additionally, heart-specific CD4+ T-cells are proposed to directly induce inflammatory myocarditis in some patients, as they have been shown to in mice models.^62^ Similar mechanisms occurring in metabotype 2 may explain its T-cell-related transcriptomic profile.

To further investigate, we performed cell-type deconvolution analysis on the DCM and control data. The results suggest differences in immune cell infiltration between metabotypes. In metabotype 1, estimated immune cell proportions were lower for monocytes and M2 macrophages, while dendritic cells were increased. Metabotype 2 was characterized by higher natural killer cells and lower neutrophils. While gene expression-based cell-type estimation has inherent limitations, including its relative nature, these findings highlight broad immune shifts between metabotypes. Interestingly, T-cell subtypes were not significantly different between metabotypes. The T-cell-related transcriptomic signature of metabotype 2 may therefore relate to activity rather than infiltration. This appears to be linked, or at least cooccurring, with metabolic effects such as dysregulation of AA metabolism and the purine salvage pathways. Interestingly, the observed increase in AMP salvage and decrease in ADP salvage in metabotype 2 may indicate a buildup of AMP. Adenosine derived from this AMP could then be transported out of the cell, where it would be expected to inhibit CD4+ T-cells, contrary to the observed transcriptomic changes.^63^ This could potentially indicate a lack of conversion of adenosine from AMP or of adenosine transport to the extracellular domain, which are not covered by our current set of metabolic tasks. This immunometabolic angle is worth investigating further in future studies.

### Limitations and Future Outlook

In this work, several technical considerations must be addressed. The effectiveness of metabolic task analysis is primarily constrained by the task list used in its quality and coverage. Defining metabolic tasks is conceptually straightforward but is fairly labor-intensive. Nevertheless, we think that well-defined tasks enhance the pipeline efficacy significantly. Moreover, these tasks can be reused across multiple data sets, increasing their utility. By improving and expanding existing task lists, such as our own, the presented pipeline could be applied to a broader range of research applications. In its current implementation; however, task activity can already be used in machine learning and multiomics approaches to identify subtypes, build classifiers and establish links between different molecular modalities. In this study, metabolic task analysis is based on transcriptomic data, using mRNA abundance as a proxy for enzyme activity. Posttranscriptional regulation, which could lead to a reduced translation of specific mRNAs, is not captured in our current framework. The same holds for posttranslational modifications such as phosphorylation or glycosylation, which could change enzyme activity. Although we demonstrate that our current framework already captures important biology, inclusion of posttranscriptional regulation and posttranslational modification data has the potential to bring results closer to what would be observed from direct metabolic flux measurements.

The clinical relevance of our investigation was also limited by the lack of detailed clinical information in the used DCM transcriptomics data set. While metabotypes did not significantly differ for any clinical variable, including left ventricular ejection fraction, they may still overlap or interact with other information that would be available to clinicians. Our results indicate a distinction in disease severity based on metabolic profiling (eg, oxidative phosphorylation, AA metabolism, and calcium pumping to the SR). If metabotypes were complementary to the clinical information in determining the disease stage, this would have important clinical value in risk prediction for patients with DCM. Furthermore, metabotypes may be useful in personalized treatments such as branched-chain AA catabolism stimulation, which has shown limited promise in animal models.^6,64^ As valine oxidation is predicted to be reduced in metabotype 2 only, branched-chain AA catabolism activation may be more beneficial in these patients. Other follow-up avenues, such as a connection between metabotypes and insulin sensitivity or outcomes cannot be examined without access to more extensive clinical data. While our results cannot therefore be readily translated to further clinical considerations yet, they demonstrate the strength of a metabolic focus in identifying subgroups, confirm existing knowledge, and provide novel insights and hypotheses.

Future research should focus on experimental validation of metabolic task analysis results. Direct flux analysis in human hearts is difficult, but targeted metabolomics and flux assays in model systems could overcome this. In rodent HF models, combined time-series transcriptomics and metabolomics could link metabolic task activity to metabolite dynamics. Targeted LC-MS/MS and enzyme assays could validate key pathways, such as purine salvage, and activities (SERCA2, RyR2, APRT), while isotope tracing in patient-derived cardiomyocytes could confirm changes in flux and substrate use. Such studies may also investigate the suitability of different animal models to mimic specific metabotypes, which would be crucial for the development of metabotype-specific therapies. From a biomarker perspective, a promising avenue is the production of carnosine, a dipeptide hypothesized to function in the heart as an antioxidant, pH buffer, and inotropic agent,^65^ which was increased in all DCM metabotypes. If confirmed, such a change may have diagnostic or prognostic implications. We also advocate for follow-up studies in subjects with varying disease severities including early stages. This would improve our understanding of cardiometabolic changes before progression to end-stage HF, when new treatments would be most effective. Finally, using data from more deeply phenotyped cohorts, we may uncover clinical markers reflecting cardiometabolism without the need for biopsies. This would also enable relating task activity to inflammatory biomarkers and immunohistological parameters, which would shine some light on the immunometabolic mechanisms hinted at by our results.

### Conclusions

Our systems biology approach shows that metabolic task analysis offers an exciting opportunity to uncover novel insights into DCM, paving the way for a deeper understanding of its progression and heterogeneity. Our results imply the presence of distinct metabotypes in end-stage DCM. Identifying the cause, impact, and progression of these metabolic shifts will be key to developing targeted treatments and discovering robust clinical biomarkers for DCM.

## ARTICLE INFORMATION

### Disclosures

None.

### Supplemental Material

Supplemental Methods

Tables S1–S5

Figures S1–S11

## Supplementary Material
