# Supplementary material for: Metabolic Task Analysis Reveals Distinct Metabotypes in End-Stage Dilated Cardiomyopathy
Source: Circ Genom Precis Med. 2026 Jul 3;19(4):e005366. doi: 10.1161/CIRCGEN.125.005366 (PMC13489769; doi:10.1161/CIRCGEN.125.005366)
Supplement: Supplementary file 1 [file hcg-19-e005366-s001.pdf]

Supplemental material

Supplemental Methods

Selection criteria for the GTEx tissue retained for analysis

Tissue selection balanced several considerations. First, only a handful of different tissues were selected as this validation exercise was not the primary objective of the manuscript. Second, only one "sub-tissue" was used per tissue as the objective of this in silico experiment was to meaningfully distinguish between tissues rather than within them. Finally, each chosen tissue was selected based on expected metabolic profile relative to heart tissue:

- Skeletal muscle: This tissue can be assumed to be the most similar to heart tissue than any other for most tasks, yet be distinct enough due to the very different metabolic demands placed on the heart compared to skeletal muscle;
- Liver: The liver is a very metabolically active organ and should be uniquely capable of performing certain metabolic functions;
- Adipose tissue: Adipose tissue was assumed to be much less active than heart tissue for ATP-production and to have high activity for fatty-acid related metabolic tasks;
- Colon: While the colon has important metabolic functions, it was assumed to be less active for most tasks than the other selected tissues.

Negative control tasks in metabolic task analysis

Artificial negative control tasks were not introduced to the task list. Indeed, tasks already existed which are not expected to occur to significant levels in the heart. Examples of such negative control tasks would be "Phenylalanine to tyrosine", which is mediated by the enzyme Phenylalanine hydroxylase that is not present in the heart, or "Starch degradation", which is also not expected to occur in the heart[28]. Both of these tasks showed zero to low activity in all MAGNet samples, being considered active in 0% and 2% of MAGNet samples, respectively. Importantly, determining whether a metabolic function should be absent in a given tissue should be done with careful consideration to avoid mistakes due to our incomplete understanding of tissue-specific human metabolism.

Supplemental Tables

Supplemental table 1: Summary characteristics of the controls and DCM subjects in the MaGNet dataset.

Characteristic	**DCM**, N = 164*	**NF**, N = 160*	**p-value**†
Age	54 (47, 59)	57 (51, 65)	0.001
Sex			0.015
Female	64 (39%)	84 (53%)	
Male	100 (61%)	76 (48%)	
Ethnicity			<0.001
Afr. American	75 (46%)	42 (26%)	
Caucasian	89 (54%)	118 (74%)	
Diabetes	39 (24%)	37 (23%)	0.9
Hypertension	77 (47%)	98 (61%)	0.010

*Median (IQR); n (%)

†Wilcoxon rank sum test; Pearson's Chi-squared test

Supplemental table 2: Summary of changes made to the Human-GEM version 1.13.0. and of the cardiac-specific metabolic tasks added to the merged task list.

**Change from Human-GEM 1.13.0**	**Reason for change**
Modified metabolite identifier nomenclature to fit Cobratoolbox standards	Improves usability of the model with Cobratoolbox-derived functions such as *CheckMetabolicTasks.*
Added sarcoplasmic reticulum as a compartment (compartment [s])	It is necessary to implement sarcoplasmic calcium pumps and channels.
Added sarcoplasmic calcium as metabolite (MAM01413[s])	A unique version of every metabolite must be created for every compartment of the model that it can enter.
Added the RyR sarcoplasmic calcium channel as a reaction with RyR1 and RyR2 as related genes (MAR20001)	Allows for the exit of Ca2+ from the sarcoplasm.
Added the Ca2+ ATPase calcium pump with the ATP2A 1:3 as related genes (MAR20002)	Allows for the active transport of Ca2+ into the sarcoplasm.
**Tasks added to the merged list**	**Task description**
Task 349: Carnosine synthesis from beta-alanine and histidine	Takes as input 1 unit of histidine, beta alanine and ATP. Returns as output 1 unit of AMP, protons, diphosphate, and carnosine. This task represents the activity of carnosine synthase 1 (CARNS1).
Task 350: Calcium pumping to SR	Takes as input 1 unit of ATP and water, and two units of calcium (cytoplasm). Returns as output 1 unit of ADP, protons, phosphate, and two units of calcium (sarcoplasm). This tasks represents the activity of ATP2A1, ATP2A2, and ATP2A3.
Task 351: Calcium flux through RyR2	Takes as input 1 unit of sarcoplasmic calcium and returns as output 1 unit of cytosolic calcium. This tasks represents the activity of the RyR channel.

Task 352: Contraction	Takes as input 1 unit of the myosin light chain and ATP. Returns as output 1 unit of activated myosin light chain and ADP. This tasks represents the activation of myosin.

Supplemental table 3: Summary of changes made to Cobratoolbox and Cellfie functions.

Function (origin)	Changes to code	Reason for change
essentialRxnsTasks (COBRA toolbox/RAVEN toolbox)	Forcing flux minimization regardless of model parameters	In some cases, flux was maximized rather than minimized, leading to absurd essential reactions
essentialRxnsTasks (COBRA toolbox/RAVEN toolbox)	Introduced a relaxation parameter	Avoids edge-cases caused by numerical instability in large models (flux-balance analysis relies on estimations with small but finite margins or error). This prevents many absurd essential reactions.
CheckMetabolicTasks (Cobratoolbox)	Allowing for the use of our modified *essentialRxnsTasks* function	Self-explanatory.
CellFie	Introducing the possibility to run CellFie in parallel	Allows the use of larger datasets which would be too computationally expensive if single-threaded.
CellFie	Introducing the possibility to skip detailed scoring	The construction of detailed scores (not used in this study) was memory-inefficient, rendering large datasets unusable.

Supplemental table 4: Tasks of interest focused upon in analysis.

Task description	Reason for inclusion
Aerobic rephosphorylation of ATP from glucose	Task concerns energy production from a specific substrate. Essential reactions largely cover glycolysis, the Krebbs cycle, and oxidative phosphorylation.
Aerobic rephosphorylation of ATP from a fatty acid	Task concerns energy production from a specific substrate. Essential reactions largely cover beta oxidation, the Krebbs cycle, and oxidative phosphorylation.
Anaerobic rephosphorylation of ATP	Task concerns energy production from a specific substrate. Essential reactions cover glycolysis well.

ADP salvage from Adenosine	ADP salvage could be meaningful in the context of an energy-deprived heart. All essential reactions are sensible.
ADP salvage from Hypoxanthine	ADP salvage could be meaningful in the context of an energy-deprived heart. All essential reactions are sensible
Release of glucose from Glycogen	Availability to use glycogen may be important. Essential reactions cover the correct pathway.
Asparagine degradation	Closer inspection revealed that ATP production from asparagine should occur through deamination, which requires H2O. H2O is missing from the task as defined. This H2O requirement forces the model to use some odd essential reactions. However, most essential reactions do cover the correct pathway, and we believe the oddities present should not impact results.
Serine degradation	Amino acid metabolism could be important in DCM. Essential reactions for this task are sensible.
Beta-Alanine degradation	Amino acid metabolism could be important in DCM. Essential reactions for this task are sensible.
Gluconeogenesis from Glutamine	Amino acid metabolism could be important in DCM. Essential reactions for this task are sensible.
Glucose to lactate conversion	Task concerns energy production from a specific substrate. Essential reactions cover glycolysis well.
Malate to pyruvate conversion	The Krebs cycle is a crucial component of cellular energy production. Essential reactions for this task are sensible.
Synthesis of fructose-6-phosphate from erythrose-4-phosphate (HMP shunt)	May be interesting as it may show diverging metabolism from glycolysis
Synthesis of ribose-5-phosphate	May be interesting as it may show diverging metabolism from glycolysis
Link between glyoxylate metabolism and pentose phosphate pathway (Xylulose to glycolate)	May be interesting as it may show diverging metabolism from glycolysis
Tyrosine to acetoacetate and fumarate	Amino acid metabolism could be important in DCM. Essential reactions for this task are sensible.
Valine to succinyl-coA	Amino acid metabolism could be important in DCM. Essential reactions for this task are sensible.
Cholesterol synthesis	Cholesterol synthesis may give insights into lipid metabolism. Essential reactions for this task are sensible.
Synthesis of ubiquinone from tyrosine	May reflect OXPHOS impact, reasonable essential reactions.
AMP salvage from adenine	Interesting insights into energy turnover, correct essential reactions
IMP salvage from hypoxanthine	Interesting insights into energy turnover, correct essential reactions
Calcium pumping to SR	Important measure of cardiac homeostasis.
Calcium flux through RyR2	Important measure of cardiac homeostasis.
Contraction	Important measure of cardiac homeostasis.

*Supplemental table 5: Fold changes between controls and clusters for all tasks. * q-value < 0.05, ** q-value < 0.01, *** q-value < 0.001. Bold highlights absolute fold change above 1.1 or below -1.1 (10% difference) in addition to significance. Met. = Metabotype.*

Task	FC DCM vs Controls	FC Met. 1 vs Controls	FC Met. 2 vs Controls	FC Met. 2 vs Met. 1
Task_1: Aerobic rephosphorylation of ATP from glucose	-1.01	-1.02**	1.01	1.02**
Task_2: Aerobic rephosphorylation of ATP from a fatty acid	1.02*	1.01	1.03**	1.02*
Task_3: Aerobic rephosphorylation of GTP	1.00	-1.01	1.03*	1.03**
Task_4: Aerobic rephosphorylation of CTP	1.00	-1.01	1.03*	1.04**
Task_5: Aerobic rephosphorylation of UTP	1.00	-1.01	1.03*	1.04**
Task_6: Anaerobic rephosphorylation of ATP	1.00	-1.02	1.04**	1.06***
Task_7: Anaerobic rephosphorylation of GTP	1.00	-1.02	1.04**	1.06***
Task_8: Anaerobic rephosphorylation of CTP	1.00	-1.02	1.04**	1.06***
Task_9: Anaerobic rephosphorylation of UTP	1.00	-1.02	1.04**	1.06***
Task_10: ATP de novo synthesis	-1.03***	-1.04***	-1.01	1.03**
Task_11: CTP de novo synthesis	-1.02*	-1.03*	-1.02	1.00
Task_12: GTP de novo synthesis	-1.07***	-1.07***	-1.07***	-1.02*
Task_13: UTP de novo synthesis	-1.02	-1.02	-1.01	1.00
Task_14: dATP de novo synthesis	-1.08***	-1.07***	**-1.11*** **	-1.05***
Task_15: dCTP de novo synthesis	-1.02*	-1.03*	-1.02	1.00
Task_16: dGTP de novo synthesis	-1.07***	-1.07***	-1.08***	-1.02*
Task_17: dTTP de novo synthesis	-1.01	-1.03*	1.01	1.03
Task_18: ADP salvage from Adenosine	-1.05**	-1.02	**-1.11*** **	-1.09***
Task_19: ADP salvage from Hypoxanthine	1.04	1.04	1.04	1.00
Task_20: dTTP salvage from Thymine	-1.05	**-1.18*** **	**1.13** **	**1.33*** **
Task_21: Aerobic reduction of NAD+	-1.01	-1.03**	1.01	1.04***
Task_22: Aerobic reduction of NADP+	1.01	-1.02	1.05*	1.07***
Task_23: Gluconeogenesis from Lactate	-1.01	-1.05***	1.05***	1.09***
Task_24: Gluconeogenesis from Glycerol	-1.00	-1.05***	1.06***	**1.11*** **
Task_25: Gluconeogenesis from Alanine	-1.01	-1.05***	1.05***	1.09***
Task_26: Gluconeogenesis from Lactate and optionally fatty acid	1.00	-1.01	1.03**	1.03***
Task_27: Gluconeogenesis from Glycerol and optionally fatty acid	-1.01	-1.03**	1.02	1.04***
Task_28: Gluconeogenesis from Alanine and fatty acid	-1.02*	-1.03***	1.00	1.03*
Task_29: Storage of glucose in Glycogen	NA	NA	NA	NA

Task_30: Release of glucose from Glycogen	1.00	-1.00	1.01	1.00
Task_31: Fructose degradation	-1.01	-1.02**	1.00	1.02**
Task_32: Galactose degradation	-1.01	-1.02**	1.01	1.02**
Task_33: UDP-glucose de novo synthesis	-1.09***	-1.06***	**-1.14*** **	-1.08***
Task_34: UDP-galactose de novo synthesis	1.07*	1.10**	1.04	-1.06
Task_35: UDP-glucuronate de novo synthesis	-1.09***	-1.08***	**-1.11*** **	-1.04**
Task_36: GDP-L-fucose de novo synthesis	1.02	1.00	1.05	1.03
Task_37: GDP-mannose de novo synthesis	1.03	1.04	1.03	-1.04
Task_38: UDP-N-acetyl D-galactosamine de novo synthesis	-1.09***	-1.10***	-1.08***	1.02
Task_39: CMP-N-acetylneuraminate de novo synthesis	-1.10***	**-1.11*** **	-1.09***	1.02
Task_40: N-Acetylglucosamine de novo synthesis	-1.06***	-1.08***	-1.02	1.05**
Task_41: Glucuronate de novo synthesis	-1.09***	-1.07***	**-1.11*** **	-1.04**
Task_42: Alanine de novo synthesis	-1.03***	-1.05***	-1.00	1.04***
Task_43: Arginine de novo synthesis	-1.02***	-1.04***	-1.00	1.03***
Task_44: Asparagine de novo synthesis	-1.04***	-1.05***	-1.03*	1.02*
Task_45: Aspartate de novo synthesis	-1.02*	-1.04***	1.00	1.03**
Task_46: Glutamate de novo synthesis	-1.03***	-1.05***	-1.01	1.03***
Task_47: Glycine de novo synthesis	-1.02**	-1.03***	-1.00	1.03***
Task_48: Glutamine de novo synthesis	-1.05***	-1.06***	-1.03*	1.03**
Task_49: Proline de novo synthesis	-1.02	-1.03***	1.01	1.03**
Task_50: Serine de novo synthesis	-1.01	-1.02**	1.01	1.02**
Task_51: Histidine de novo synthesis	NA	NA	NA	NA
Task_52: Histidine uptake	-1.00	-1.09*	**1.12**	**1.24** **
Task_53: Isoleucine de novo synthesis	NA	NA	NA	NA
Task_54: Isoleucine uptake	-1.00	-1.09*	**1.12**	**1.24** **
Task_55: Leucine de novo synthesis	NA	NA	NA	NA
Task_56: Leucine uptake	-1.00	-1.09*	**1.12**	**1.24** **
Task_57: Lysine de novo synthesis	NA	NA	NA	NA
Task_58: Lysine uptake	**-1.18** **	**-1.24*** **	-1.09	**1.37** **
Task_59: Methionine de novo synthesis	NA	NA	NA	NA
Task_60: Methionine uptake	-1.05***	-1.06**	-1.03*	1.00
Task_61: Phenylalanine de novo synthesis	NA	NA	NA	NA
Task_62: Phenylalanine uptake	**1.27**	**1.36**	**1.14**	**-1.38**

Task_63: Threonine de novo synthesis	NA	NA	NA	NA
Task_64: Threonine uptake	1.06	-1.09*	**1.28**	**1.38****
Task_65: Tryptophan de novo synthesis	NA	NA	NA	NA
Task_66: Tryptophan uptake	**1.27**	**1.36**	**1.14**	**-1.38**
Task_67: Valine de novo synthesis	NA	NA	NA	NA
Task_68: Valine uptake	-1.00	-1.09*	**1.12**	**1.24****
Task_69: Cysteine de novo synthesis from glucose	NA	NA	NA	NA
Task_70: Cysteine de novo synthesis from AAs	**-1.35*****	**-1.34*****	**-1.37*****	-1.02
Task_71: Cystine de novo synthesis	NA	NA	NA	NA
Task_72: Tyrosine de novo synthesis	NA	NA	NA	NA
Task_73: Tyrosine de novo synthesis	1.09***	1.07***	**1.12*****	1.04*
Task_74: Homocysteine de novo synthesis	NA	NA	NA	NA
Task_75: Homocysteine de novo synthesis	**-1.34*****	**-1.32*****	**-1.38*****	-1.03
Task_76: beta-Alanine de novo synthesis	-1.03***	-1.04***	-1.02	1.01
Task_77: Alanine degradation	-1.07***	-1.06***	-1.08***	-1.04**
Task_78: Arginine degradation	-1.04***	-1.05***	-1.03**	1.01
Task_79: Asparagine degradation	**-1.12*****	-1.09***	**-1.17*****	-1.10***
Task_80: Aspartate degradation	-1.06***	-1.07***	-1.06***	-1.01
Task_81: Cysteine degradation	-1.06***	-1.05***	-1.06***	-1.02
Task_82: Glutamate degradation	-1.07***	-1.07***	-1.06***	1.00
Task_83: Glycine degradation	1.02	-1.05*	**1.14*****	**1.19*****
Task_84: Histidine degradation	-1.07***	-1.07***	-1.06***	1.00
Task_85: Isoleucine degradation	-1.06***	-1.06***	-1.05***	-1.00
Task_86: Glutamine degradation	-1.06***	-1.06***	-1.05***	1.00
Task_87: Leucine degradation	-1.05***	-1.05***	-1.06***	-1.02
Task_88: Lysine degradation	-1.06***	-1.06***	-1.05***	-1.00
Task_89: Methionine degradation	-1.06***	-1.07***	-1.04**	1.02*
Task_90: Phenylalanine degradation	-1.06***	-1.06***	-1.05***	1.00
Task_91: Proline degradation	-1.05***	-1.06***	-1.05***	1.00
Task_92: Serine degradation	-1.03	-1.07***	1.04*	**1.12*****
Task_93: Threonine degradation	-1.06***	-1.06***	-1.06***	-1.01
Task_94: Tryptophan degradation	-1.05***	-1.06***	-1.04***	1.00
Task_95: Tyrosine degradation	-1.06***	-1.06***	-1.07***	-1.03*

Task_96: Valine degradation	-1.05***	-1.06***	-1.05***	-1.00
Task_97: Homocysteine degradation	**-1.42*** **	**-1.47*** **	**-1.35*** **	1.07*
Task_98: Ornithine degradation	-1.05***	-1.06***	-1.05***	1.00
Task_99: beta-Alanine degradation	-1.06***	-1.05***	-1.07***	-1.03
Task_100: Urea from alanine	-1.04***	-1.04***	-1.03*	1.01
Task_101: Urea from glutamine	-1.04***	-1.04***	-1.03**	-1.00
Task_102: Creatine de novo synthesis	**1.43*** **	**1.39*** **	**1.50*** **	1.09
Task_103: Heme de novo synthesis	-1.04***	-1.02**	-1.06***	-1.04***
Task_104: PC de novo synthesis	-1.04***	-1.04***	-1.03**	1.01
Task_105: PE de novo synthesis	-1.04***	-1.05***	-1.02*	1.02**
Task_106: PS de novo synthesis	-1.03***	-1.04***	-1.02*	1.01
Task_107: PI de novo synthesis	-1.03***	-1.04***	-1.01	1.02*
Task_108: Cardiolipin de novo synthesis	-1.02***	-1.03***	-1.01	1.01
Task_109: SM de novo synthesis	-1.04***	-1.04***	-1.03***	1.00
Task_110: Ceramide de novo synthesis	-1.03***	-1.04***	-1.02**	1.01
Task_111: Lactosylceramide de novo synthesis	-1.03***	-1.03***	-1.02**	-1.00
Task_112: CoA de novo synthesis	1.03*	-1.01	**1.10*** **	**1.11*** **
Task_113: NAD de novo synthesis	**-1.43*** **	**-1.31*** **	**-1.66*** **	**-1.25*** **
Task_114: NADP de novo synthesis	-1.02**	-1.04***	-1.00	1.03*
Task_115: FAD de novo synthesis	**-1.16*** **	**-1.15*** **	**-1.17*** **	-1.02
Task_116: Thioredoxin de novo synthesis	NA	NA	NA	NA
Task_117: THF de novo synthesis	-1.08**	-1.03	**-1.15*** **	**-1.11*** **
Task_118: Pyridoxal-P de novo synthesis	-1.04*	**-1.12*** **	1.06	**1.17*** **
Task_119: Acetoacetate de novo synthesis	-1.05***	-1.06***	-1.03*	1.02
Task_120: (R)-3-Hydroxybutanoate de novo synthesis	-1.04***	-1.05***	-1.03	1.02
Task_121: Farnesyl-PP de novo synthesis	-1.01	-1.08***	1.09***	**1.16*** **
Task_122: Lauric acid de novo synthesis	-1.03***	-1.03***	-1.02	1.01
Task_123: Tridecylic acid de novo synthesis	-1.03***	-1.04***	-1.02	1.02*
Task_124: Myristic acid de novo synthesis	-1.03***	-1.03***	-1.02	1.01
Task_125: 9E-tetradecenoic acid de novo synthesis	-1.03***	-1.03***	-1.02	1.01
Task_126: 7Z-tetradecenoic acid de novo synthesis	-1.03***	-1.03***	-1.02	1.01
Task_127: Physeteric acid de novo synthesis	-1.03***	-1.03***	-1.02	1.01
Task_128: Pentadecylic acid de novo synthesis	-1.03***	-1.04***	-1.02	1.02*

Task_129: Palmitate de novo synthesis	-1.03***	-1.04***	-1.02	1.02*
Task_130: Palmitolate de novo synthesis	-1.03**	-1.04***	-1.01	1.02*
Task_131: 7-palmitoleic acid de novo synthesis	-1.03***	-1.03***	-1.02	1.01
Task_132: Margaric acid de novo synthesis	-1.03***	-1.04***	-1.02	1.02*
Task_133: 10Z-heptadecenoic acid de novo synthesis	-1.03***	-1.04***	-1.02	1.02*
Task_134: 9-heptadecylenic acid de novo synthesis	-1.03***	-1.04***	-1.02	1.02*
Task_135: Stearate de novo synthesis	-1.01	-1.02	1.01	1.02*
Task_136: 13Z-octadecenoic acid de novo synthesis	-1.03***	-1.03***	-1.02	1.01
Task_137: cis-vaccenic acid de novo synthesis	-1.03**	-1.04***	-1.01	1.02*
Task_138: Oleate de novo synthesis	-1.01	-1.02	1.01	1.02*
Task_139: Elaidate de novo synthesis	-1.03**	-1.04***	-1.01	1.02*
Task_140: 7Z-octadecenoic acid de novo synthesis	-1.03***	-1.03***	-1.02	1.01
Task_141: 6Z,9Z-octadecadienoic acid de novo synthesis	-1.03***	-1.03***	-1.02	1.01
Task_142: Nonadecylic acid de novo synthesis	-1.03***	-1.04***	-1.02	1.02*
Task_143: Eicosanoate de novo synthesis	-1.03***	-1.03***	-1.02	1.01
Task_144: 13Z-Eicosenoic acid de novo synthesis	-1.03***	-1.03***	-1.02	1.01
Task_145: cis-gondoic acid de novo synthesis	-1.03***	-1.03***	-1.02	1.01
Task_146: 9-Eicosenoic acid de novo synthesis	-1.03***	-1.03***	-1.02	1.01
Task_147: 8,11-Eicosadienoic acid de novo synthesis	-1.03***	-1.03***	-1.02	1.01
Task_148: Mead acid de novo synthesis	-1.03***	-1.04***	-1.02	1.01
Task_149: Henicosanoic acid de novo synthesis	-1.03***	-1.04***	-1.02	1.02*
Task_150: Behenic acid de novo synthesis	-1.03***	-1.03***	-1.02	1.01
Task_151: cis-erucic acid de novo synthesis	-1.03***	-1.03***	-1.02	1.01
Task_152: cis-cetoleic acid de novo synthesis	-1.04***	-1.05***	-1.04**	1.01
Task_153: Tricosanoic acid de novo synthesis	-1.03***	-1.04***	-1.01	1.02*
Task_154: Lignocerate de novo synthesis	-1.03***	-1.03***	-1.02	1.00
Task_155: Nervonic acid de novo synthesis	-1.04***	-1.05***	-1.04**	1.00
Task_156: Cerotic acid de novo synthesis	-1.03***	-1.03***	-1.02	1.00
Task_157: Ximenic acid de novo synthesis	-1.04***	-1.05***	-1.04**	1.00
Task_158: Linolenate de novo synthesis	NA	NA	NA	NA
Task_159: Stearidonic acid de novo synthesis	-1.01	-1.02**	1.02	1.04***
Task_160: omega-3-Arachidonic acid de novo synthesis	-1.01	-1.02**	1.00	1.02*

Task_161: EPA de novo synthesis	-1.01	-1.03**	1.02	1.04***
Task_162: DPA de novo synthesis	-1.01	-1.02**	1.00	1.02*
Task_163: 9Z,12Z,15Z,18Z,21Z-TPA de novo synthesis	-1.01	-1.02**	1.00	1.02**
Task_164: 6Z,9Z,12Z,15Z,18Z,21Z-THA de novo synthesis	-1.01	-1.02**	1.00	1.02**
Task_165: DHA de novo synthesis	-1.01	-1.02*	1.01	1.03**
Task_166: 11Z,14Z,17Z-eicosatrienoic acid de novo synthesis	-1.02	-1.03**	1.00	1.02*
Task_167: 13,16,19-docosatrienoic acid de novo synthesis	-1.02	-1.03**	1.00	1.02*
Task_168: 10,13,16,19-docosatetraenoic acid de novo synthesis	-1.01	-1.02**	1.00	1.02*
Task_169: 12,15,18,21-tetracosatetraenoic acid de novo synthesis	-1.01	-1.02**	1.00	1.02*
Task_170: Linoleate de novo synthesis	NA	NA	NA	NA
Task_171: gamma-Linolenate de novo synthesis	**-1.36*** **	**-1.44*** **	**-1.24** **	**1.11**
Task_172: Dihomo-gamma-linolenate de novo synthesis	1.01	-1.02	1.05**	1.07***
Task_173: Arachidonate de novo synthesis	-1.03**	-1.04***	-1.01	1.02**
Task_174: Adrenic acid de novo synthesis	-1.01	-1.02**	1.01	1.03**
Task_175: 9Z,12Z,15Z,18Z-TTA de novo synthesis	-1.01	-1.02**	1.01	1.03**
Task_176: 6Z,9Z,12Z,15Z,18Z-TPA de novo synthesis	-1.01	-1.02**	1.01	1.03**
Task_177: 4Z,7Z,10Z,13Z,16Z-DPA de novo synthesis	-1.01	-1.02**	1.00	1.02*
Task_178: 11Z,14Z-eicosadienoic acid de novo synthesis	-1.01	-1.02**	1.00	1.02*
Task_179: 13Z,16Z-docosadienoic acid de novo synthesis	-1.01	-1.02**	1.00	1.02*
Task_180: 10,13,16-docosatriynoic acid de novo synthesis	-1.01	-1.02**	1.01	1.02**
Task_181: Lauric acid complete oxidation	-1.05***	-1.04***	-1.05***	-1.02
Task_182: Tridecylic acid complete oxidation	-1.05***	-1.04***	-1.06***	-1.03
Task_183: Myristic acid complete oxidation	-1.05***	-1.04***	-1.05***	-1.02
Task_184: 9E-tetradecenoic acid complete oxidation	-1.05***	-1.04***	-1.06***	-1.02
Task_185: 7Z-tetradecenoic acid complete oxidation	-1.05***	-1.04***	-1.05***	-1.02
Task_186: Physeteric acid complete oxidation	-1.05***	-1.04***	-1.05***	-1.02
Task_187: Pentadecylic acid complete oxidation	-1.05***	-1.04***	-1.06***	-1.02
Task_188: Palmitate complete oxidation	-1.04***	-1.04***	-1.05***	-1.02

Task_189: Palmitolate complete oxidation	-1.04***	-1.04***	-1.05***	-1.02
Task_190: 7-palmitoleic acid complete oxidation	-1.04***	-1.04***	-1.05***	-1.02
Task_191: Margaric acid complete oxidation	-1.05***	-1.04***	-1.05***	-1.02
Task_192: 10Z-heptadecenoic acid complete oxidation	-1.05***	-1.04***	-1.05***	-1.02
Task_193: 9-heptadecylenic acid complete oxidation	-1.05***	-1.04***	-1.05***	-1.02
Task_194: Stearate complete oxidation	-1.04***	-1.04***	-1.05***	-1.02
Task_195: 13Z-octadecenoic acid complete oxidation	-1.04***	-1.04***	-1.05***	-1.02
Task_196: cis-vaccenic acid complete oxidation	-1.04***	-1.04***	-1.05***	-1.02
Task_197: Oleate complete oxidation	-1.04***	-1.04***	-1.05***	-1.02
Task_198: Elaidate complete oxidation	-1.04***	-1.04***	-1.05***	-1.02
Task_199: 7Z-octadecenoic acid complete oxidation	-1.04***	-1.04***	-1.05***	-1.02
Task_200: 6Z,9Z-octadecadienoic acid complete oxidation	-1.04***	-1.04***	-1.05***	-1.02
Task_201: Nonadecylic acid complete oxidation	-1.05***	-1.04***	-1.05***	-1.02
Task_202: Eicosanoate complete oxidation	-1.04***	-1.04***	-1.05***	-1.02
Task_203: 13Z-Eicosenoic acid complete oxidation	-1.04***	-1.04***	-1.05***	-1.02
Task_204: cis-gondoic acid complete oxidation	-1.04***	-1.04***	-1.05***	-1.02
Task_205: 9-Eicosenoic acid complete oxidation	-1.04***	-1.04***	-1.05***	-1.02
Task_206: 8,11-Eicosadienoic acid complete oxidation	-1.04***	-1.04***	-1.05***	-1.02
Task_207: Mead acid complete oxidation	-1.04***	-1.04***	-1.05***	-1.02
Task_208: Henicosanoic acid complete oxidation	-1.04***	-1.04***	-1.05***	-1.01
Task_209: Behenic acid complete oxidation	-1.04***	-1.04***	-1.05***	-1.02
Task_210: cis-erucic acid complete oxidation	-1.04***	-1.04***	-1.05***	-1.02
Task_211: cis-cetoleic acid complete oxidation	-1.04***	-1.04***	-1.05***	-1.02
Task_212: Tricosanoic acid complete oxidation	-1.05***	-1.04***	-1.06***	-1.02
Task_213: Lignocerate complete oxidation	-1.05***	-1.04***	-1.06***	-1.02
Task_214: Nervonic acid complete oxidation	-1.04***	-1.04***	-1.05***	-1.02
Task_215: Cerotic acid complete oxidation	-1.04***	-1.04***	-1.05***	-1.02
Task_216: Ximenic acid complete oxidation	-1.04***	-1.04***	-1.05***	-1.02
Task_217: Linolenate complete oxidation	-1.04***	-1.04***	-1.05***	-1.02
Task_218: Stearidonic acid complete oxidation	-1.04***	-1.04***	-1.05***	-1.02
Task_219: omega-3-Arachidonic acid complete oxidation	-1.04***	-1.04***	-1.04***	-1.02
Task_220: EPA complete oxidation	-1.04***	-1.04***	-1.05***	-1.02

Task_221: DPA complete oxidation	-1.04***	-1.04***	-1.05***	-1.02
Task_222: 9Z,12Z,15Z,18Z,21Z-TPA complete oxidation	-1.04***	-1.04***	-1.05***	-1.02
Task_223: 6Z,9Z,12Z,15Z,18Z,21Z-THA complete oxidation	-1.04***	-1.04***	-1.05***	-1.02
Task_224: DHA complete oxidation	-1.04***	-1.04***	-1.05***	-1.02
Task_225: 11Z,14Z,17Z-eicosatrienoic acid complete oxidation	-1.04***	-1.04***	-1.05***	-1.02
Task_226: 13,16,19-docosatrienoic acid complete oxidation	-1.04***	-1.04***	-1.05***	-1.02
Task_227: 10,13,16,19-docosatetraenoic acid complete oxidation	-1.04***	-1.04***	-1.05***	-1.02
Task_228: 12,15,18,21-tetracosatetraenoic acid complete oxidation	-1.05***	-1.04***	-1.06***	-1.02
Task_229: Linoleate complete oxidation	-1.04***	-1.04***	-1.05***	-1.02
Task_230: gamma-Linolenate complete oxidation	-1.04***	-1.04***	-1.05***	-1.02
Task_231: Dihomo-gamma-linolenate complete oxidation	-1.04***	-1.04***	-1.05***	-1.02
Task_232: Arachidonate complete oxidation	-1.04***	-1.04***	-1.05***	-1.02
Task_233: Adrenic acid complete oxidation	-1.04***	-1.04***	-1.04**	-1.01
Task_234: 9Z,12Z,15Z,18Z-TTA complete oxidation	-1.04***	-1.04***	-1.04***	-1.01
Task_235: 6Z,9Z,12Z,15Z,18Z-TPA complete oxidation	-1.04***	-1.04***	-1.05***	-1.02
Task_236: 4Z,7Z,10Z,13Z,16Z-DPA complete oxidation	-1.04***	-1.04***	-1.05***	-1.02
Task_237: 11Z,14Z-eicosadienoic acid complete oxidation	-1.04***	-1.04***	-1.05***	-1.02
Task_238: 13Z,16Z-docosadienoic acid complete oxidation	-1.04***	-1.04***	-1.05***	-1.03
Task_239: 10,13,16-docosatriynoic acid complete oxidation	-1.04***	-1.04***	-1.05***	-1.02
Task_240: Triacylglycerol de novo synthesis	-1.04***	-1.05***	-1.02*	1.03**
Task_241: Glycocholate de novo synthesis and excretion	-1.03*	-1.05***	-1.00	1.03
Task_242: Glycochenodeoxycholate de novo synthesis and excretion	-1.02*	-1.02*	-1.02	-1.02
Task_243: Taurocholate de novo synthesis and excretion	-1.03*	-1.05***	-1.00	1.03
Task_244: Taurochenodeoxycholate de novo synthesis and excretion	1.03	1.00	1.07***	1.06***
Task_245: PAPS de novo synthesis	**-1.13*****	**-1.11*****	**-1.15*****	-1.06*
Task_246: PAPS degradation	-1.04***	-1.05***	-1.03**	1.02

Task_247: SAM de novo synthesis	-1.10***	-1.05***	**-1.18***	**-1.12***
Task_248: GSH de novo synthesis	-1.05***	-1.04***	-1.06***	-1.04*
Task_249: Bilirubin conjugation	NA	NA	NA	NA
Task_250: NH3 import and degradation	-1.01	-1.02**	1.00	1.02*
Task_251: Ethanol import and degradation	-1.03**	-1.01	-1.05**	-1.04**
Task_252: Oxidative phosphorylation	1.07	1.02	**1.16***	1.07
Task_253: Oxidative decarboxylation	**-1.47***	**-1.36***	**-1.68***	**-1.31***
Task_254: Krebs cycle NADH	1.00	1.01	-1.01	-1.02
Task_255: Ubiquinol-to-proton	1.02	1.00	1.05*	1.05**
Task_256: Ubiquinol-to-ATP	1.01	-1.00	1.03*	1.04**
Task_257: Biosynthesis of Vitamin C	NA	NA	NA	NA
Task_259: Reactive oxygen species detoxification	-1.08**	-1.03	**-1.18***	**-1.15***
Task_260: Presence of the thioredoxin system through the thioredoxin reductase activity	**-1.30***	**-1.35***	**-1.24***	1.05
Task_261: Inosine monophosphate synthesis	-1.10***	-1.08***	**-1.13***	-1.06***
Task_262: GMP salvage from guanine	1.03*	1.04**	1.01	-1.03
Task_263: ATP generation from ions	NA	NA	NA	NA
Task_264: Degradation of adenine to urate	**-1.11***	**-1.12***	**-1.11***	-1.01
Task_265: Degradation of guanine to urate	-1.06***	-1.06***	-1.05*	1.02
Task_266: Degradation of cytosine	1.01	-1.01	1.06*	1.06**
Task_267: Degradation of uracil	1.01	-1.01	1.06*	1.06**
Task_268: Gluconeogenesis from pyruvate	-1.01	-1.05***	1.05***	1.09***
Task_269: Gluconeogenesis from Glutamine	-1.02	1.02	-1.09***	**-1.11***
Task_270: Glucose to lactate conversion	1.00	-1.02	1.04**	1.06***
Task_271: Malate to pyruvate conversion	**-1.11***	-1.03	**-1.25***	**-1.23***
Task_272: Synthesis of erythrose-4-phosphate from fructose-6-phosphate	**-1.16***	**-1.21***	-1.10**	**1.12**
Task_273: Synthesis of ribose-5-phosphate	-1.10***	-1.10***	**-1.10***	-1.02
Task_274: Synthesis of lactose	**-1.70***	**-1.48***	**-2.20***	**-1.93*
Task_275: Fructose to glucose conversion	**-1.10***	**-1.12***	-1.08**	1.03
Task_276: Mannose degradation	1.06**	1.03	1.10**	1.06*
Task_278: Synthesis of acetone	-1.05***	-1.06***	-1.03*	1.02
Task_279: Synthesis of inositol	1.02	-1.04	**1.11**	**1.13***
Task_280: Inositol as input for glucuronate-xylulose pathway	1.04*	-1.01	**1.12***	**1.13***

Task_281: Synthesis of phosphatidylinositol from inositol	**1.10*** **	1.02	**1.23*** **	**1.20*** **
Task_282: Conversion of 1-phosphatidyl-1D-myo-inositol 4,5-bisphosphate to 1D-myo-inositol 1,4,5-trisphosphate	**-1.23*** **	**-1.15*** **	**-1.37*** **	**-1.25*** **
Task_283: Starch degradation	**1.18** **	**1.32*** **	-1.03	**-1.28*** **
Task_284: Link between glyoxylate metabolism and pentose phosphate pathway	1.06***	1.04**	1.09***	1.04***
Task_285: Synthesis of methylglyoxal	1.02*	1.02	1.03*	1.01
Task_286: Synthesis of alanine from glutamine	NA	NA	NA	NA
Task_287: Synthesis of arginine from glutamine	-1.03***	-1.04***	-1.03**	-1.01
Task_288: Synthesis of nitric oxide from arginine	-1.08	**-1.11***	-1.03	1.07
Task_289: Synthesis of aspartate from glutamine	-1.00	1.00	-1.01	-1.01
Task_290: Conversion of aspartate to arginine	**-1.12*** **	**-1.19*** **	-1.02	**1.14*** **
Task_291: Conversion of aspartate to beta-alanine	1.02***	1.02**	1.03***	-1.00
Task_292: Conversion of asparate to asparagine	-1.04	**-1.11***	1.06*	**1.16*** **
Task_293: Conversion of carnosine to beta-alanine	-1.03*	-1.07***	1.02	1.08***
Task_294: Beta-alanine to 3-oxopropanoate	1.04	**1.14*** **	**-1.14***	**-1.32*** **
Task_295: Synthesis of cysteine from cystine	NA	NA	NA	NA
Task_296: Synthesis of taurine from cysteine	1.08*	1.03	**1.16** **	**1.11**
Task_297: Conversion of glutamate to glutamine	**-1.32*** **	**-1.30*** **	**-1.35*** **	-1.05
Task_298: Conversion of glutamate to proline	**1.16*** **	**1.11*** **	**1.22*** **	1.09***
Task_299: Conversion of GABA into succinate	1.09***	**1.17*** **	-1.05	**-1.25*** **
Task_300: Glutaminolysis	-1.02	1.02	-1.08***	**-1.10*** **
Task_301: Conversion of glycine to pyruvate	**-1.12*** **	**-1.18*** **	-1.04*	1.09*
Task_302: Conversion of histidine to glutamate	-1.06***	-1.06***	-1.06***	1.00
Task_303: Conversion of histidine to histamine	**1.50*** **	**1.44*** **	**1.58*** **	1.10
Task_304: Conversion of leucine to acetyl-coA	1.01	1.05*	-1.05	**-1.15*** **
Task_305: Conversion of lysine to L-Saccharopine	**-1.57*** **	**-1.32*** **	**-2.23*** **	**-1.60*** **
Task_306: Conversion of lysine to L-2-aminoadipate	**1.29*** **	**1.26*** **	**1.35*** **	1.04
Task_307: Synthesis of ornithine from glutamine	1.04**	1.08***	-1.04	**-1.13*** **
Task_308: Synthesis of spermidine from ornithine	**-1.10*** **	-1.08***	**-1.14*** **	-1.06***
Task_309: Phenylalanine to phenylacetaldehyde	-1.02*	1.01	-1.07***	-1.06***
Task_310: Phenylalanine to phenylacetyl-L-glutaminate	-1.03**	-1.01*	-1.04**	-1.02*

Task_311: Phenylalanine to tyrosine	-1.00	-1.00	1.00	1.00
Task_312: Synthesis of proline from glutamine	1.07***	1.10***	1.04	-1.06**
Task_313: Synthesis of anthranilate from tryptophan	**1.19*****	1.04**	**1.41****	**1.40**
Task_314: Synthesis of kynate from tryptophan	1.01	-1.06	**1.12**	**1.17***
Task_315: Synthesis of L-kynurenine from tryptophan	1.09*	1.02	**1.21***	**1.19**
Task_316: Synthesis of N-formylanthranilate from tryptophan	-1.03*	**-1.14****	**1.13**	**1.30**
Task_317: Synthesis of quinolinate from tryptophan	**1.22*****	**1.12*****	**1.37*****	**1.22*****
Task_318: Synthesis of serotonin from tryptophan	-1.02	1.01	-1.06**	-1.07**
Task_319: Tyrosine to adrenaline	**-1.14*****	**-1.24*****	-1.02	**1.19*****
Task_320: Tyrosine to dopamine	1.00	1.00	1.01	1.00
Task_321: Tyrosine to acetoacetate and fumarate	1.03*	-1.03	**1.12*****	**1.14*****
Task_322: Valine to succinyl-coA	-1.04*	1.02	**-1.15*****	**-1.17*****
Task_323: Hydroxymethylglutaryl-CoA synthesis	-1.05***	-1.06***	-1.03	1.03*
Task_324: Cholesterol synthesis	**-1.11*****	**-1.12*****	-1.09***	1.01
Task_325: mevalonate synthesis	-1.08***	-1.02	**-1.21*****	**-1.20*****
Task_326: Glycerol-3-phosphate synthesis	**-1.22*****	**-1.14*****	**-1.35*****	**-1.19*****
Task_327: Synthesis of malonyl-coa	-1.05***	-1.06***	-1.04*	1.02
Task_328: Synthesis of palmitoyl-CoA	-1.05***	-1.06***	-1.04**	1.02
Task_329: Synthesis of thromboxane from arachidonate	**-1.18*****	**-1.30*****	-1.03	**1.25*****
Task_330: Synthesis of glucocerebroside	-1.02***	-1.03***	-1.01	1.01
Task_331: Synthesis of globoside	-1.03***	-1.04***	-1.02*	1.01
Task_332: Synthesis of ubiquinone from tyrosine	-1.01	1.00	-1.04	-1.05
Task_333: Synthesis of bilirubin	**-1.17*****	**-1.25*****	-1.08	**1.17*****
Task_334: Phosphatidyl-inositol to glucosaminyl-acylphosphatidylinositol	-1.05***	-1.03**	-1.07***	-1.04**
Task_335: Glucosaminyl-acylphosphatidylinositoll to deacylated-glycophosphatidylinositol	-1.03*	-1.00	-1.07***	-1.09***
Task_336: Degradation of n2m2nmasn	-1.01	-1.07***	1.07*	**1.13*****
Task_337: Biosynthesis of m4mpdol_U	-1.01	-1.04***	1.04***	1.07***
Task_338: Biosynthesis of g3m8masn	-1.03*	-1.03	-1.04	-1.02
Task_339: Degradation of s2l2fn2m2masn	1.03	-1.05*	**1.14*****	**1.20*****
Task_340: Biosynthesis of core2	-1.08***	-1.09***	-1.05*	1.03
Task_341: Biosynthesis of core4	-1.04*	-1.08***	1.02	**1.11****

Task_342: Biosynthesis of Tn_antigen	-1.07**	**-1.14*** **	1.02	**1.15**
Task_343: Keratan sulfate biosynthesis from O-glycan	-1.04*	-1.07***	-1.01	1.07*
Task_344: Keratan sulfate biosynthesis from O-glycan	-1.04*	-1.07***	-1.01	1.07*
Task_345: Keratan sulfate degradation	-1.00	-1.02*	1.02	1.04*
Task_346: Keratan sulfate biosynthesis from N-glycan	-1.02	-1.06***	1.04*	1.08***
Task_347: AMP salvage from adenine	1.00	**-1.21*** **	**1.29*** **	**1.53*** **
Task_348: IMP salvage from hypoxanthine	1.03*	1.04**	1.01	-1.03
Task_349: Carnosine synthesis from beta-alanine and histidine	**1.60*** **	**1.54*** **	**1.70*** **	**1.10**
Task_350: Calcium pumping to SR	**-1.23*** **	**-1.16*** **	**-1.37*** **	**-1.18*** **
Task_351: Calcium flux through RyR2	-1.07**	1.04	**-1.29*** **	**-1.34*** **
Task_352: Contraction	**-1.12*** **	**-1.11** **	**-1.14** **	-1.05

Supplemental Figures

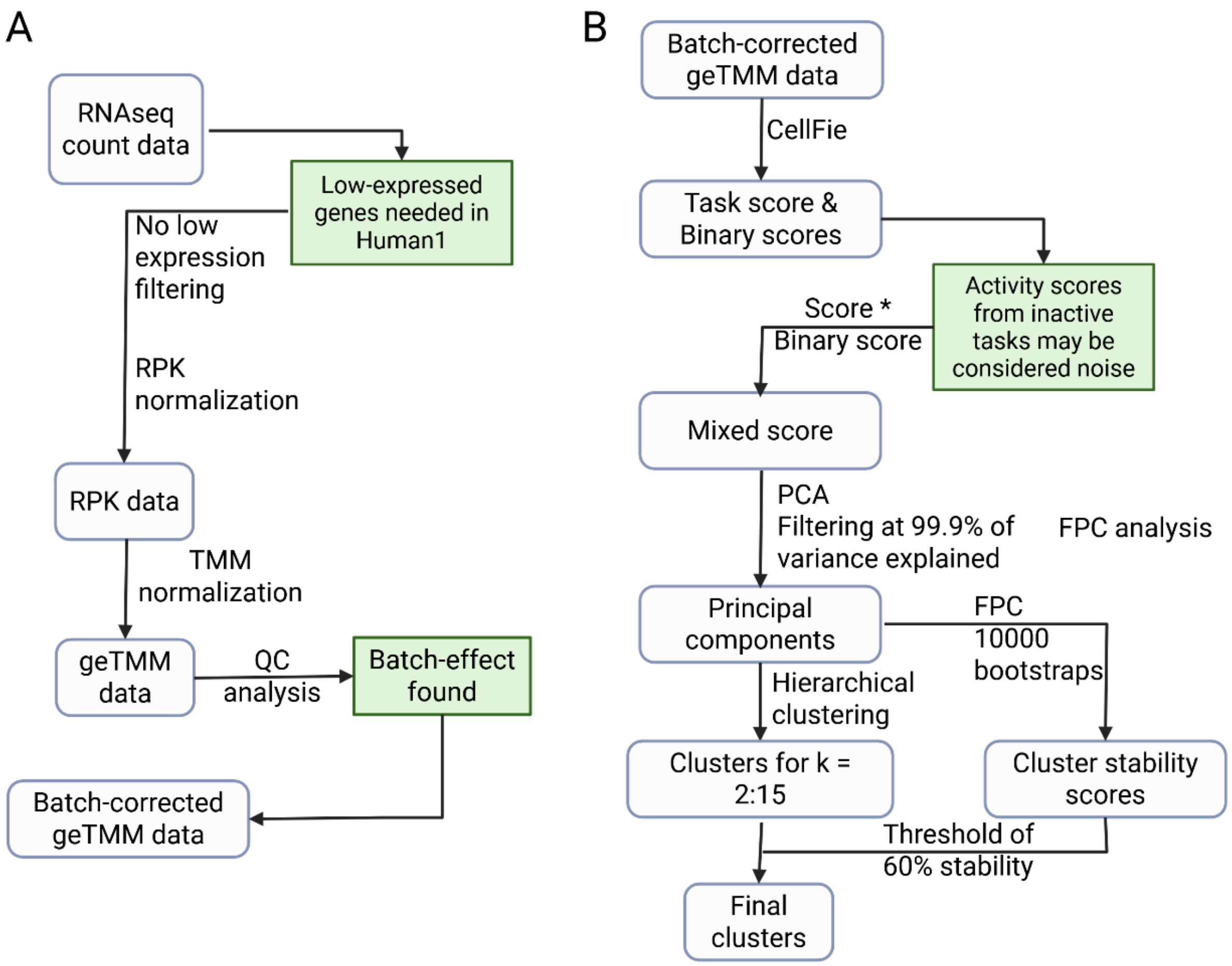

Supplemental figure 1: Flowchart of the metabotyping pipeline with data and results as white rounded boxes, actions as arrows, and decisions as green boxes. A: The RNA-seq expression data must be pre-processed. Unlike in most other

applications, low-expressed genes are not filtered out as this low expression is important information for the genome-scale metabolic model. The data must then be normalized both for length (RPK) and library size (TMM). Here, a batch effect was observed and required correction. Other confounders (RNA integrity, unrelated biological effects, etc) may be corrected in other studies. B: The pre-processed data can be used as input to the Cellfie algorithm to obtain scores and binary scores. Multiplying these results in mixed scores that serve as input to the principal component analysis. The top 99.9% of components are then used for hierarchical clustering along with the FPC algorithm to decide on the best number of clusters.

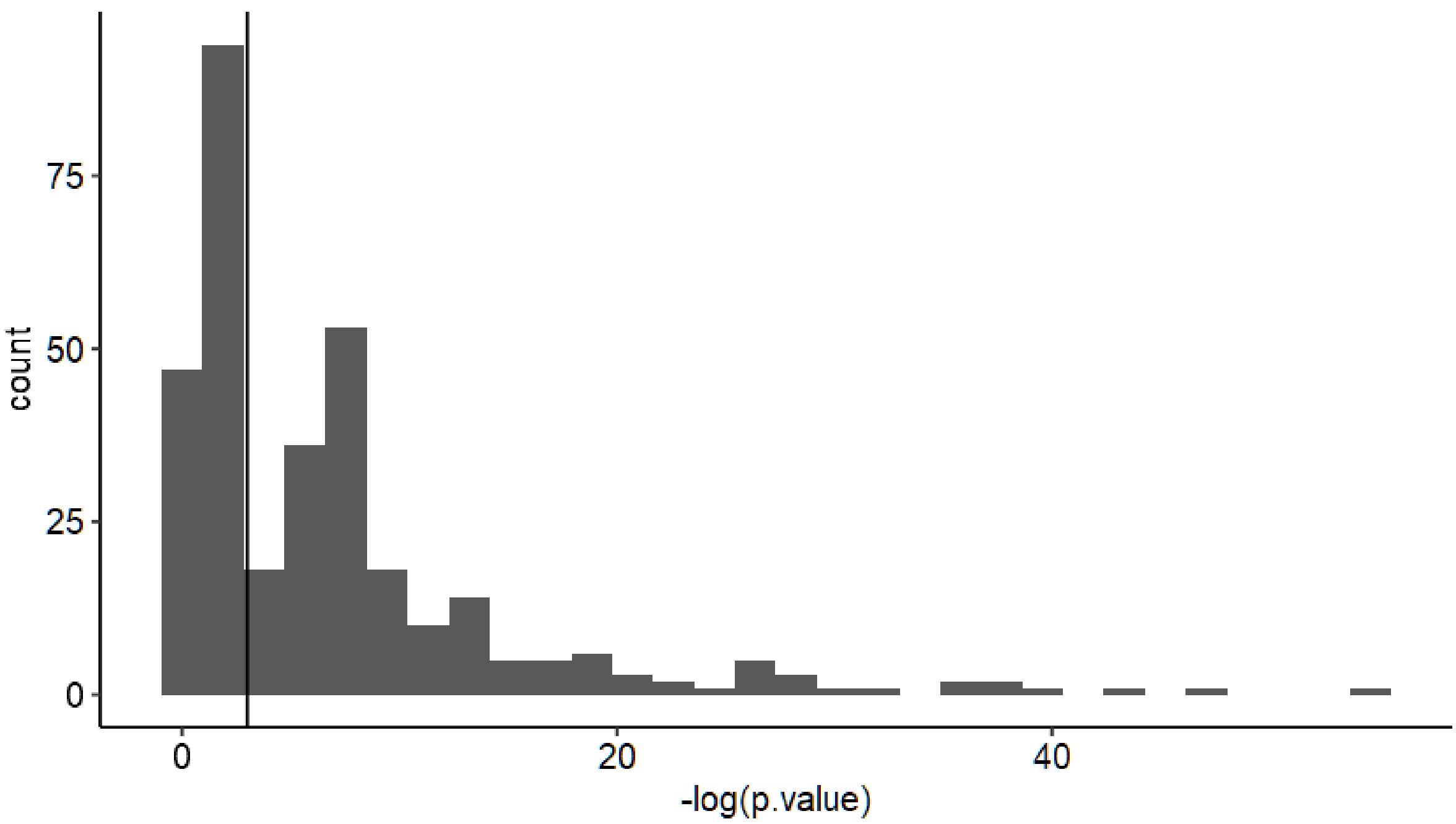

Supplemental figure 2: histogram of Shapiro-test p-values, calculated for each task from the task activity score of MaGNet control samples. Values to the left of the vertical line do not fit the normality assumption.

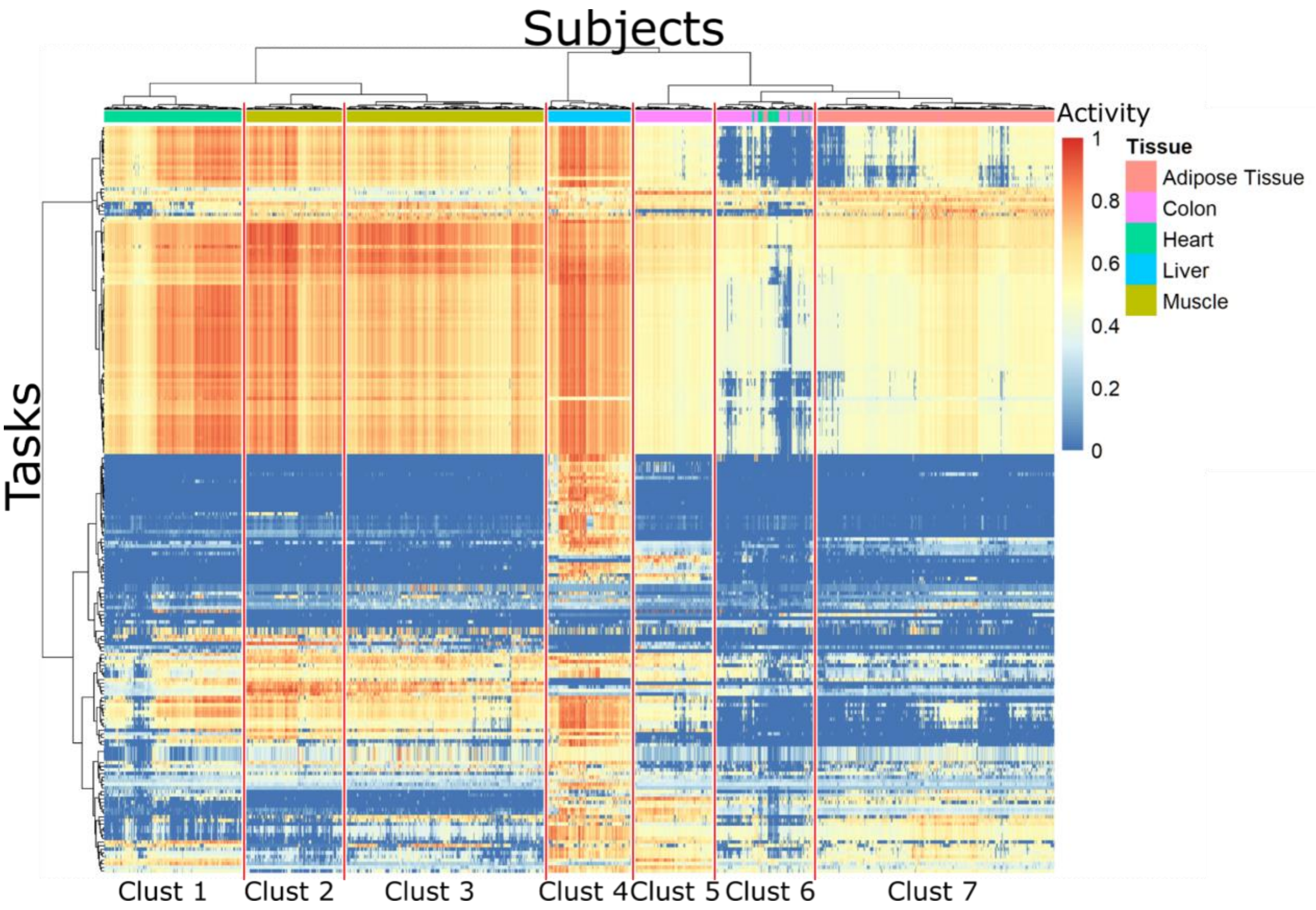

Supplemental figure 3: Heatmap of GTEx mixed activity score for the tasks included in clustering, showing the clustering.

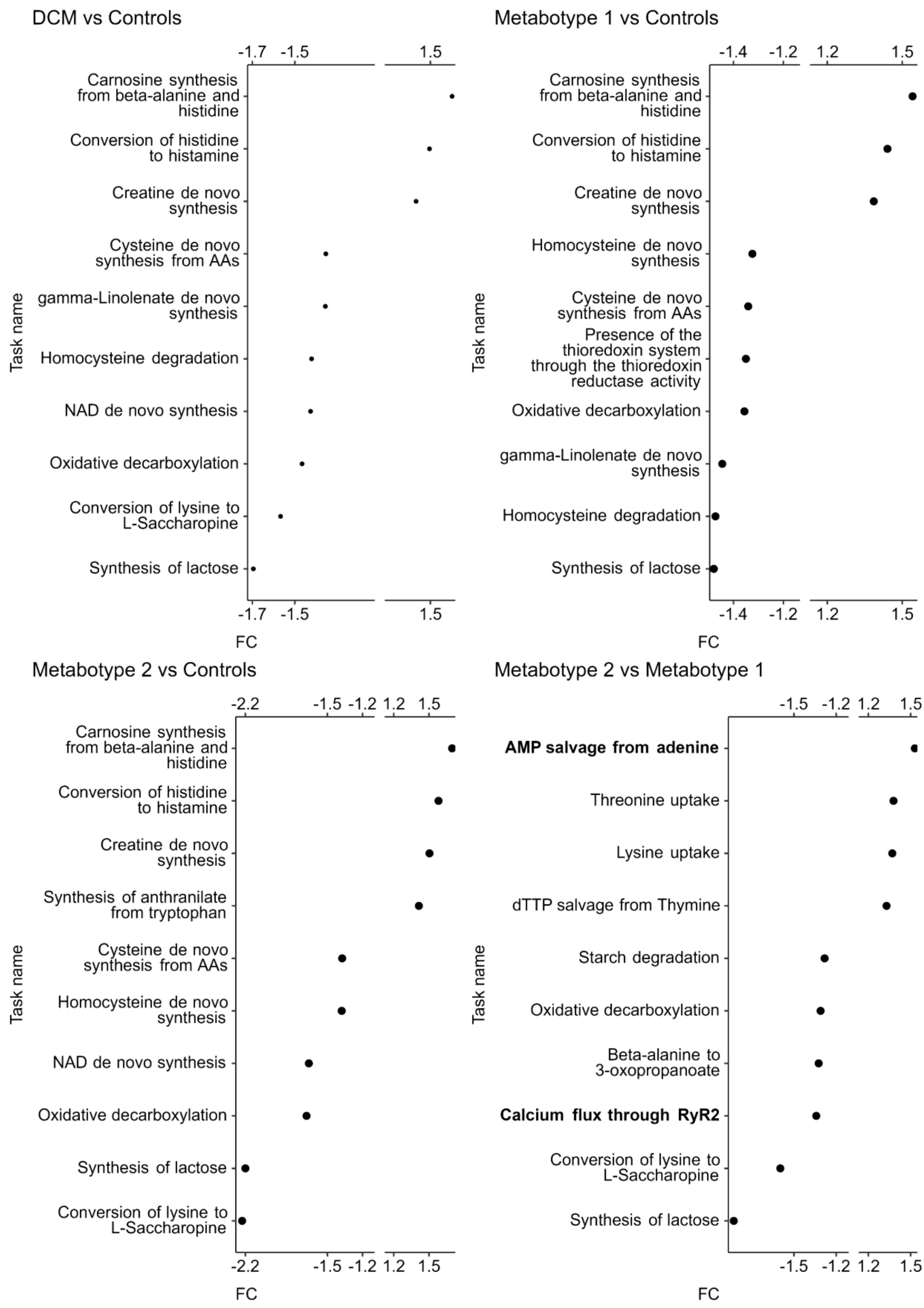

Supplemental figure 4: Fold-changes of the top 10 most different tasks (based on absolute fold-change) in each comparison made. FDR < 0.05 and absolute FC > 1.1 for all tasks presented. Task names in bold are part of the predefined tasks of interest. All of the tasks in the figure were verified to have sensible

essential reactions, although task "Synthesis of anthranilate from tryptophan" only covers the conversion of tryptophan to L-formylkynurenine.

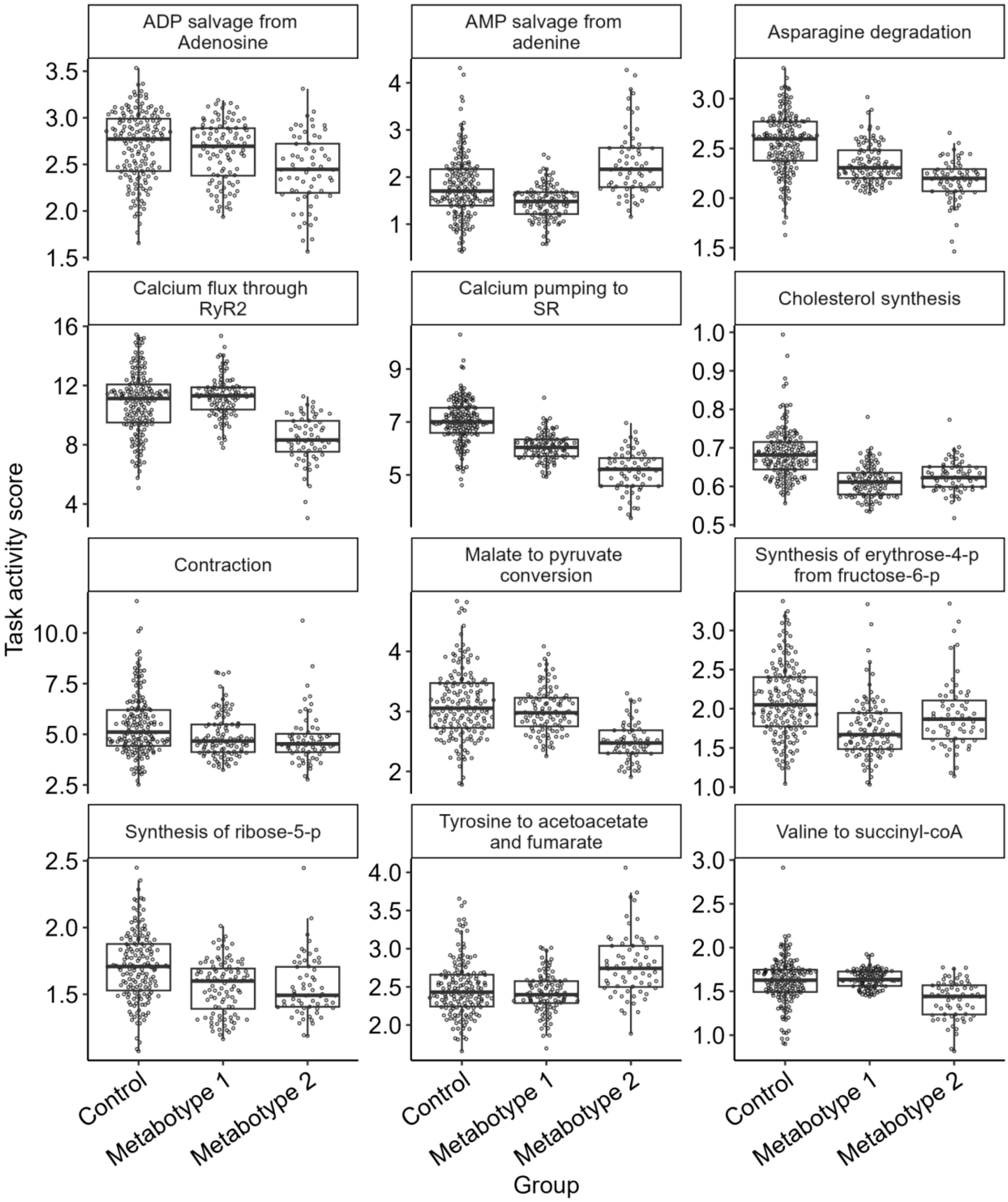

Supplemental figure 5: Boxplots showing task activity scores per group for each task of interest discussed in table 1.

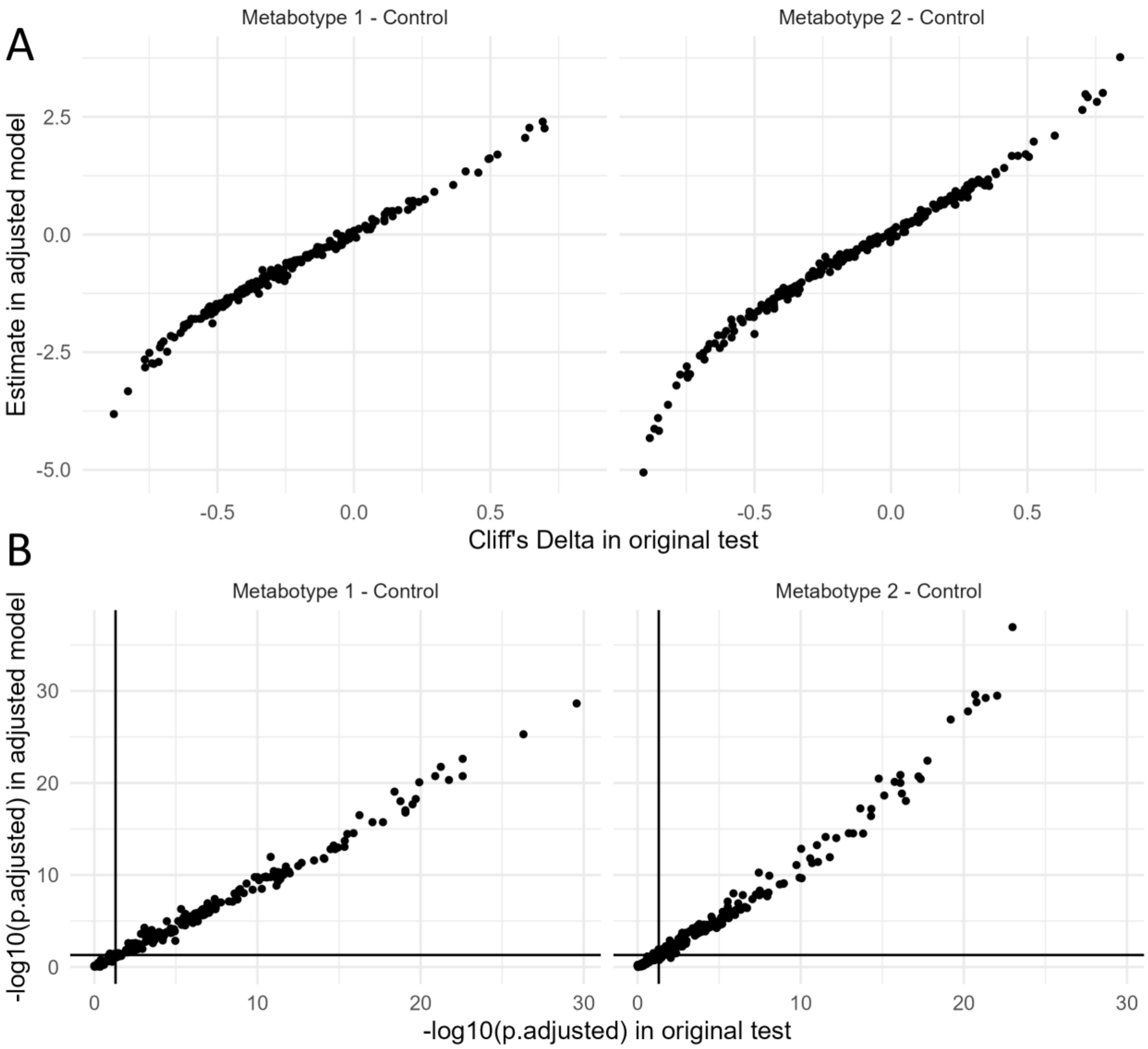

Supplemental figure 6: Comparison between effect size measures (A) and FDR-adjusted p-values (B) between the Mann-Whitney u-test and a corrected ordinal logistic regression model including age, sex, and ethnicity as covariates.

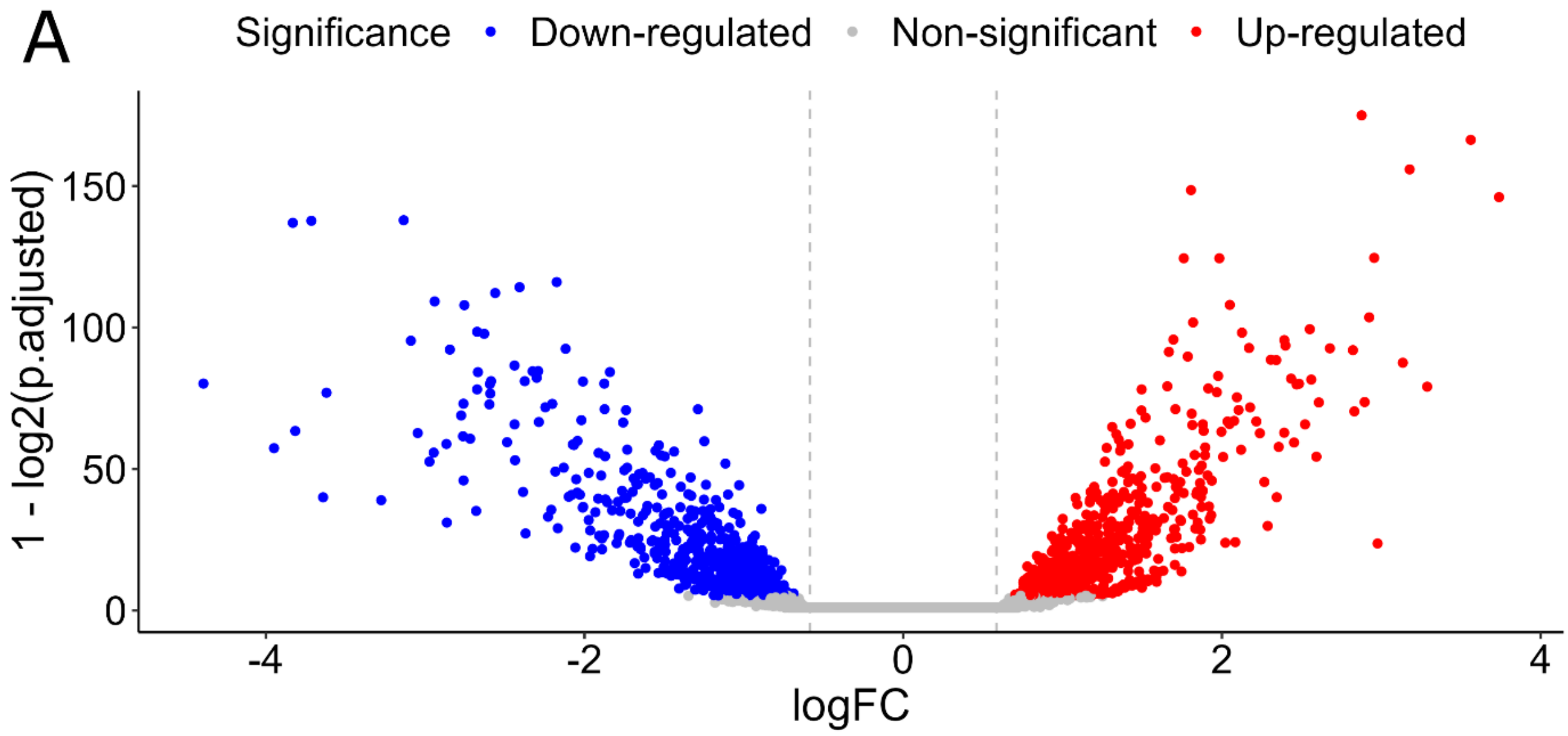

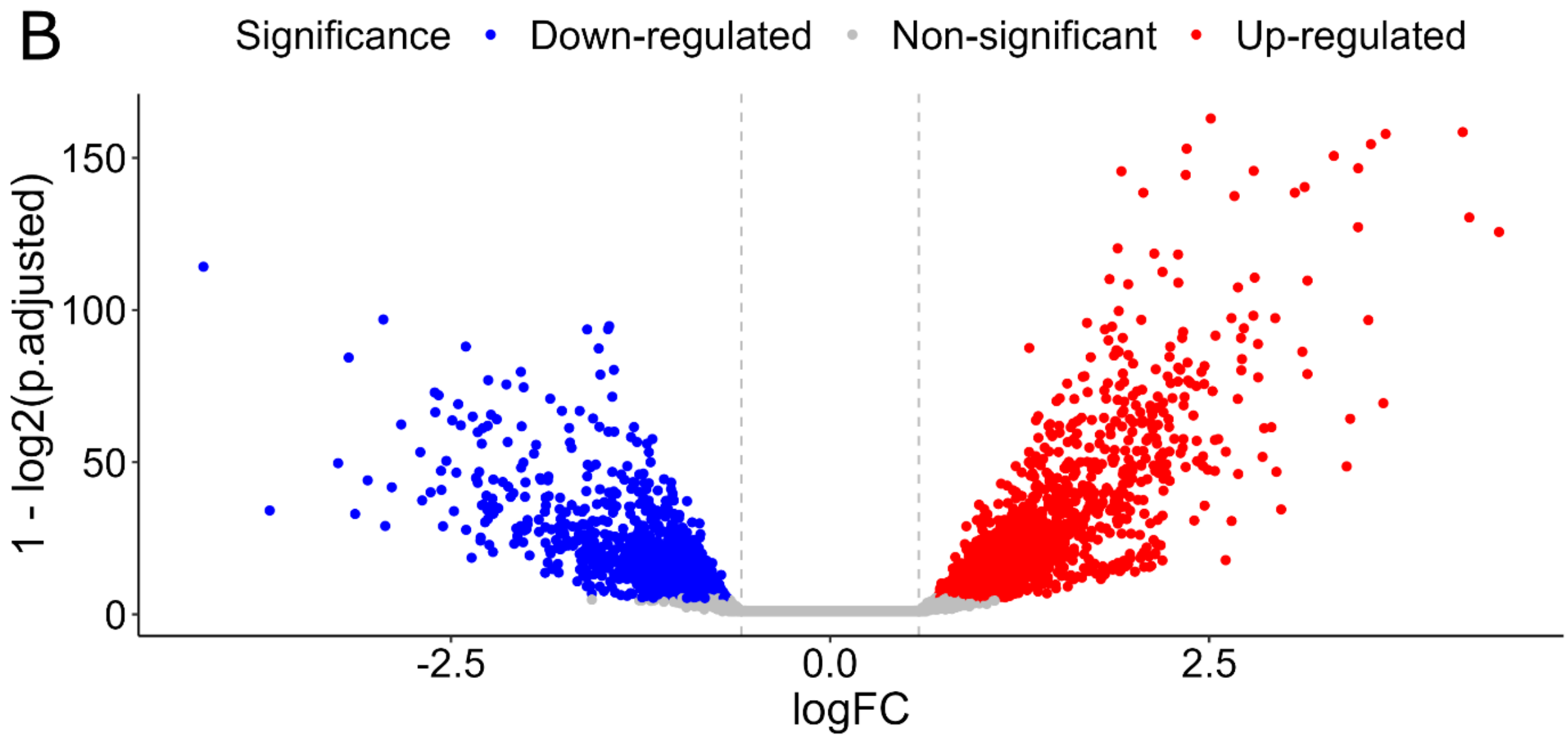

Supplemental figure 7: Volcano plot showing FDR-adjusted p-values in the differential expression analysis between controls and metabotypes 1 (A) and 2 (B).

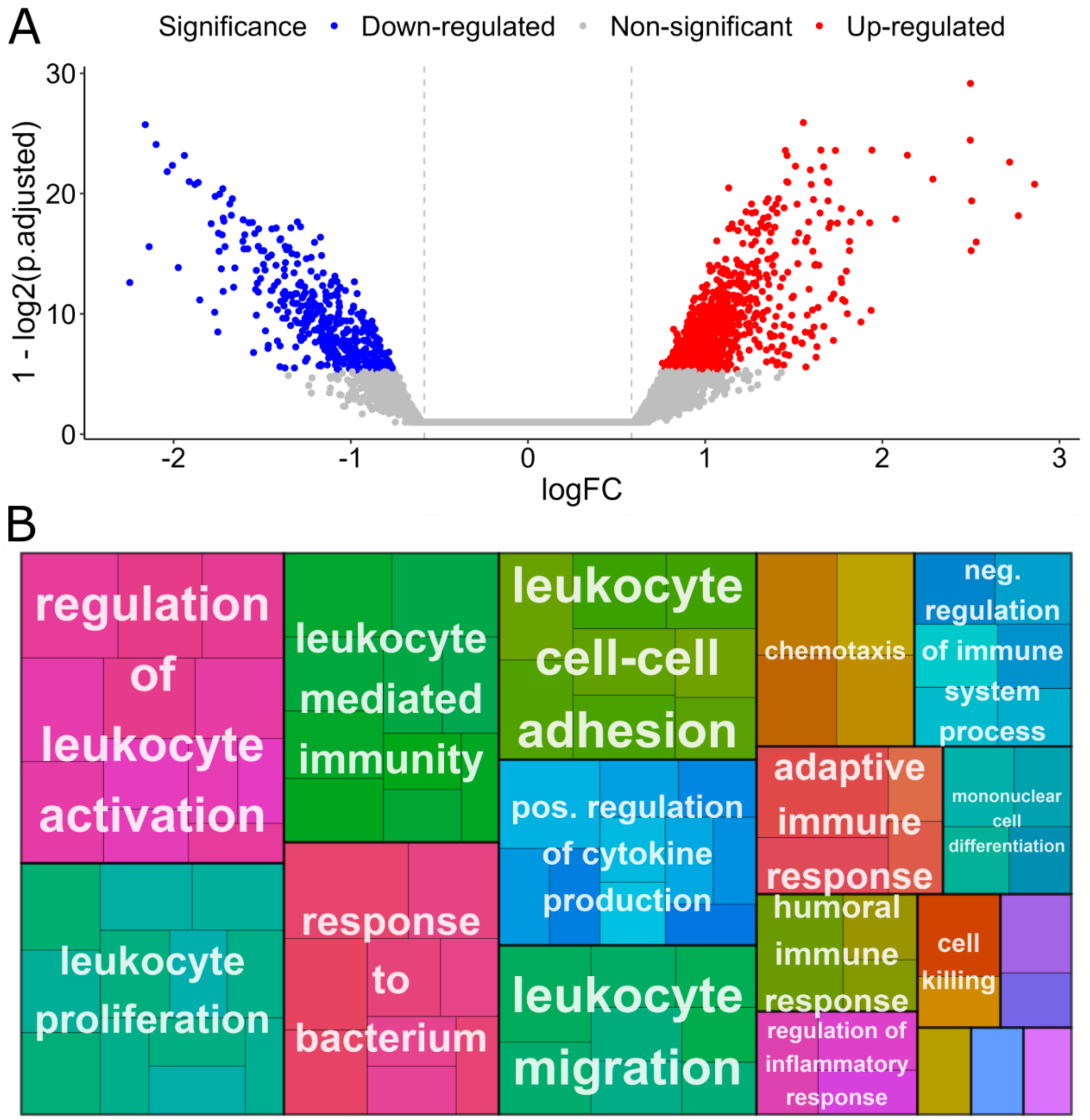

Supplemental figure 8: Volcano plot showing FDR-adjusted p-values in the differential expression analysis between metabotypes (A). Treemap plot of top 100 significantly enriched gene ontologies between metabotypes, grouped by similarity and represented by the most significant ontology in each group. Small rectangles represent individual ontologies with area relative to GSEA score. Colors assigned at random for visual contrast. (B).

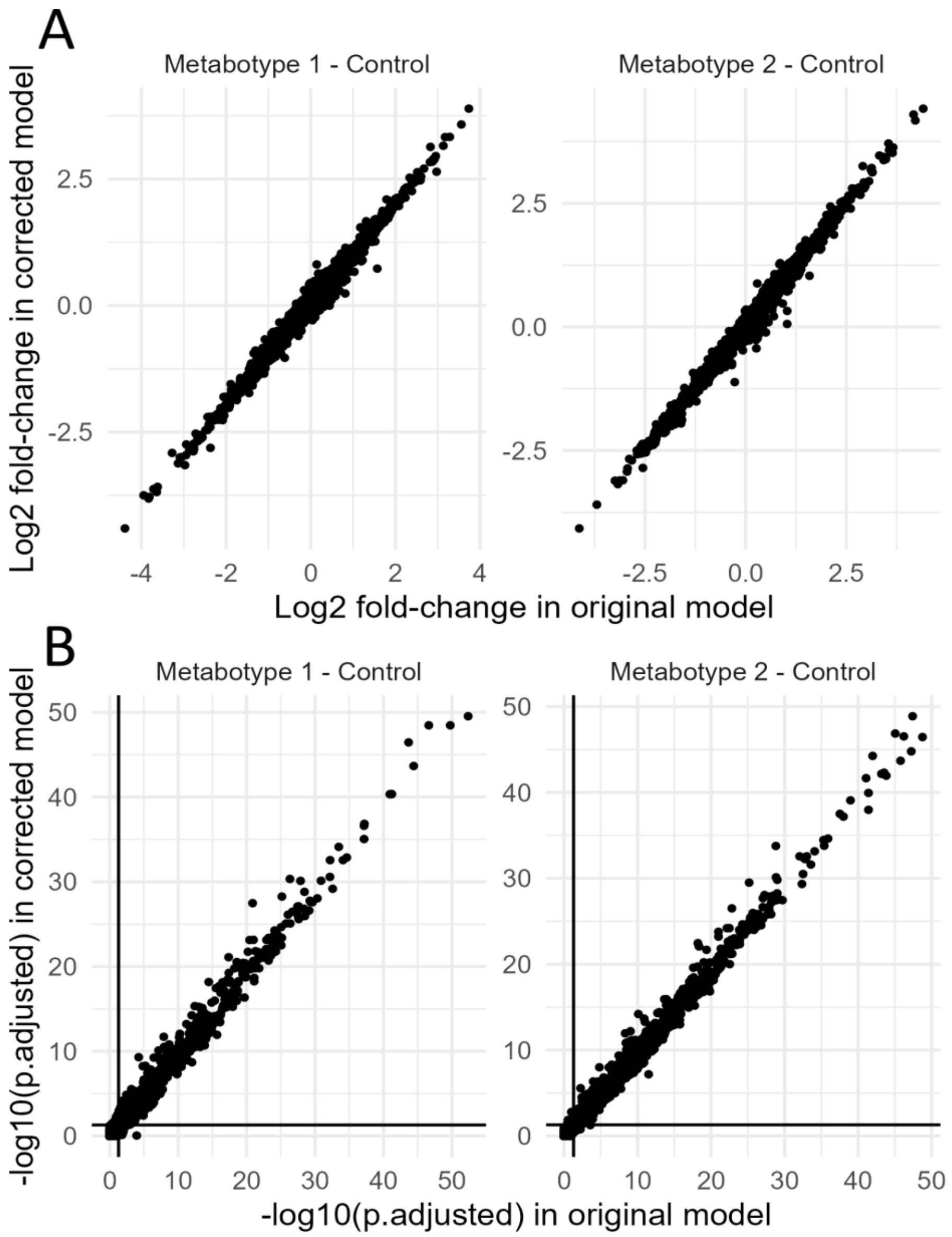

Supplemental figure 9: Comparison between Log2 Fold-changes (A) and FDR-adjusted p-values (B) between the original model and a corrected model including age, sex, and ethnicity as covariates.

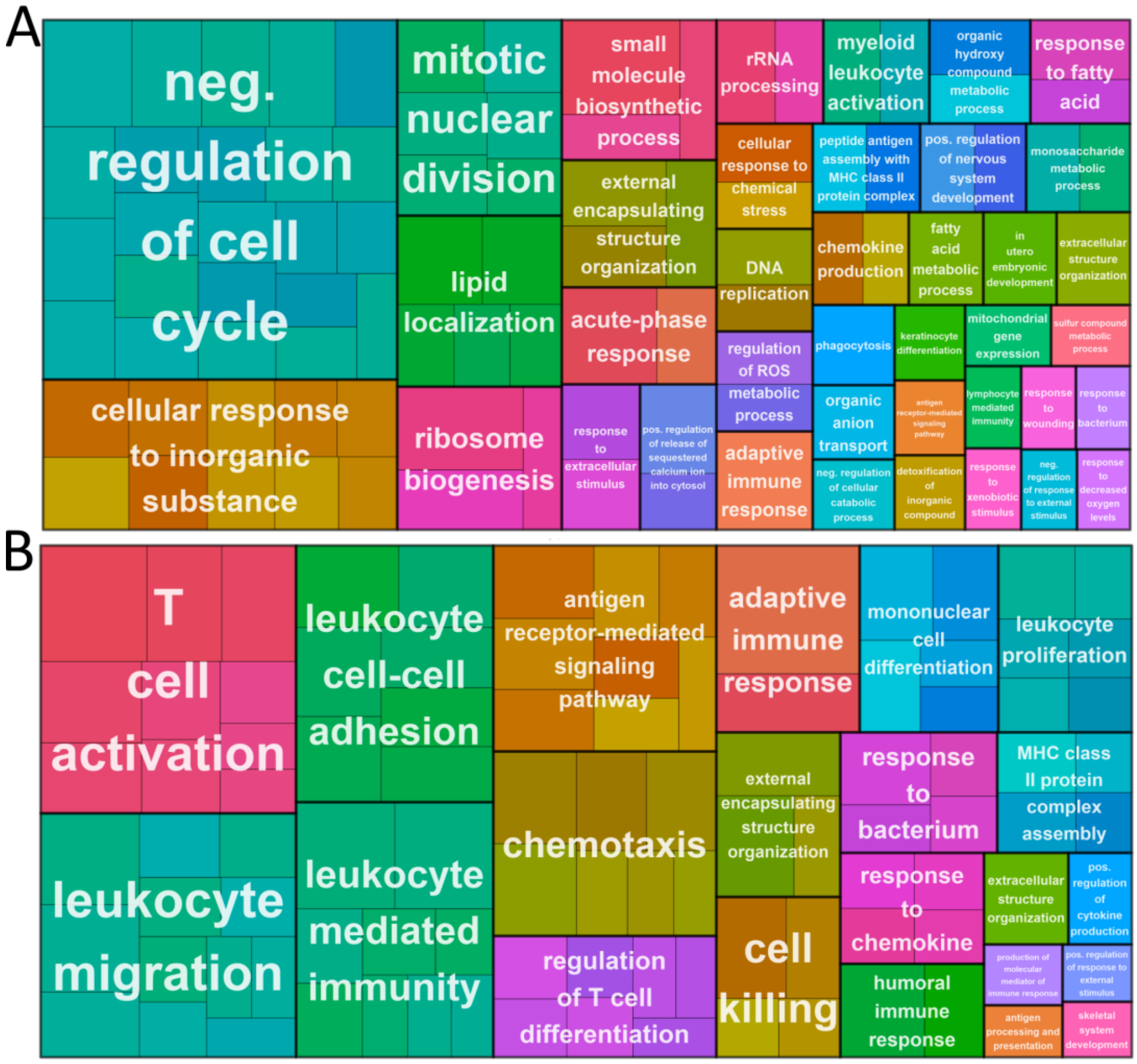

Supplemental figure 10: Treemap plot of top 100 significantly enriched gene ontologies between controls and metabotype 1 (A) or metabotype 2 (B) based on an adjusted model with age, sex, and ethnicity as covariates. Ontologies are grouped by similarity and represented by the most significant ontology in each group. Small rectangles represent individual ontologies with area relative to GSEA score. Colors assigned at random for visual contrast.

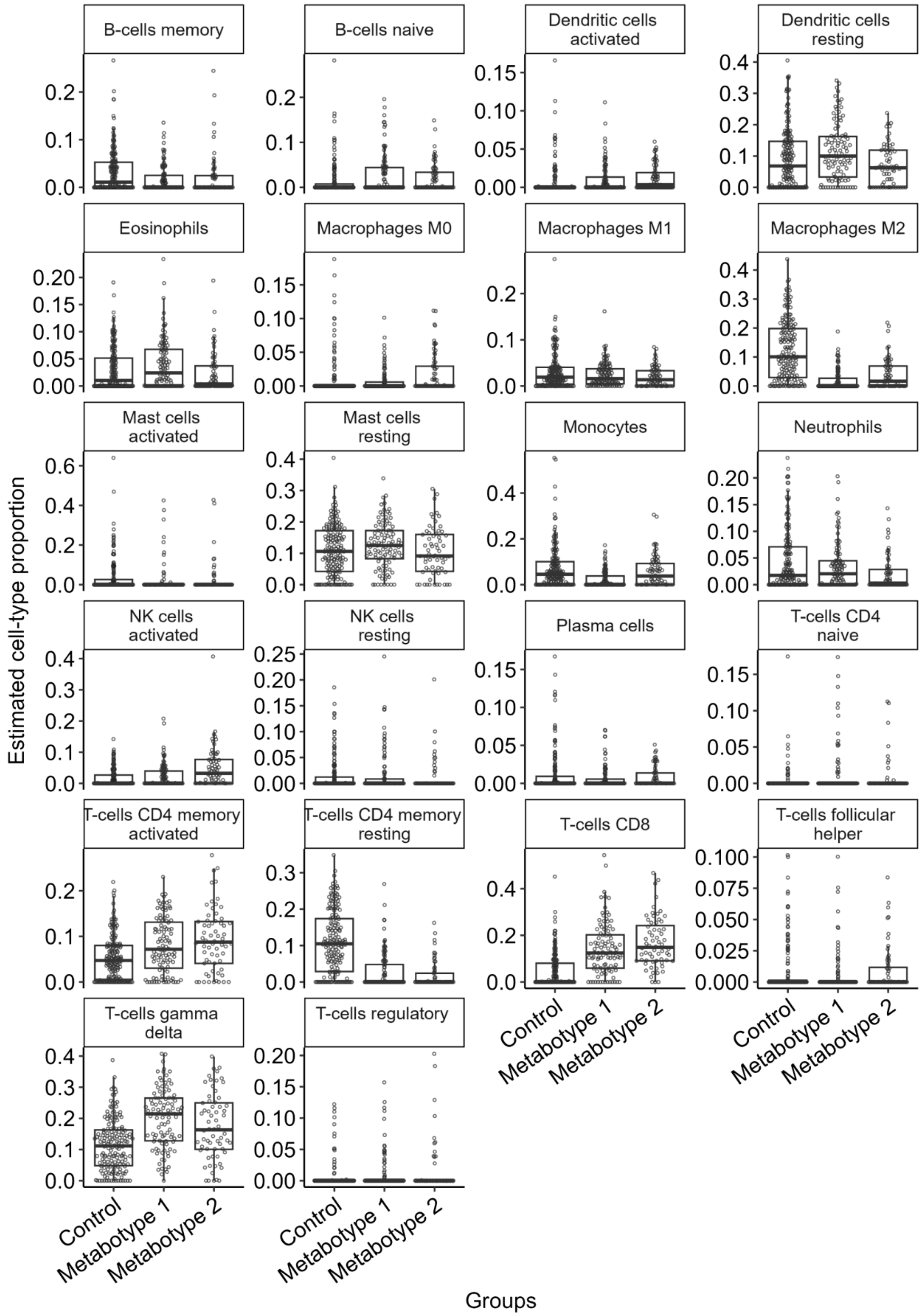

*Supplemental figure 11: Boxplots showing estimated cell-type proportions imputed using CIBERSORT in the MaGNet dataset. * p-value < 0.05, ** p-value < 0.01, *** p-value < 0.001*
